# An Eight-Gene Metabolic Prognostic Signature in Head and Neck Squamous Cell Carcinoma: Cross-Cohort Evaluation, Single-Cell Analysis, and Preliminary Functional Evaluation of PYGL and G6PD

**DOI:** 10.7150/jca.134402

**Published:** 2026-10-12

**Authors:** Wanyi Kou, Ruixin Guo, Yifan Zhang, Chensi Xu, Wenqi Sha, Yijie Chen, Yuran Lei, Ningrui Zhang, Kun Liao, Danni Liang, Yang Zhang, Zhenxing Wei, Zhenghui Wang

**Affiliations:** 1 Department of Otolaryngology-Head and Neck Surgery, Shaanxi Provincial Key Laboratory for Precision Diagnosis and Treatment of Otorhinolaryngology, The Second Affiliated Hospital of Xi'an Jiaotong University, Xi'an 710004, China; 2 Department of Otolaryngology-Head and Neck Surgery, Luoyang Central Hospital Affiliated to Zhengzhou University

**Keywords:** machine learning, HNSCC, metabolism-related prognostic score, single-cell RNA sequencing, prognostic model.

## Abstract

**Background:**

Head and neck squamous cell carcinoma (HNSCC) is the most prevalent pathological subtype of head and neck malignancies. Although metabolic reprogramming contributes to malignant progression and adaptation within the tumor microenvironment in HNSCC, its biological and prognostic implications remain incompletely understood.

**Objective:**

To develop and evaluate a metabolism-related prognostic score for head and neck squamous cell carcinoma (HNSCC) across multiple cohorts, identify its cellular sources at single-cell resolution, and perform preliminary *in vitro* functional evaluation of PYGL and G6PD.

**Methods:**

Metabolism-related differentially expressed genes were screened in The Cancer Genome Atlas Head and Neck Squamous Cell Carcinoma cohort (TCGA-HNSC). A total of 506 patients with survival data were divided into a training set (n = 355) and an internal test set (n = 151) at a 7:3 ratio. Training-only repeated nested cross-validation benchmarked 126 algorithmic configurations. Survival-SVM had the highest mean out-of-fold C-index, whereas an eight-gene LASSO + StepCox model was retained for interpretability and cross-cohort transportability. External cohort evaluation of overall survival (OS) was performed in GSE65858 (n = 270), while GSE117973 (n = 77), which used disease-specific survival (DSS), was treated as an exploratory cohort. By integrating 54,163 single cells from the GSE150321 and GSE172577 datasets, analyses, including inferCNV, 12 predefined metabolic modules, CellChat, and scTenifoldKnk, were conducted to investigate the cellular origins of the risk score and candidate regulatory networks; additionally, PYGL- and G6PD-targeting siRNAs were evaluated for their effects on target protein expression and wound closure *in vitro*.

**Results:**

We developed a metabolic prognostic model that included HPRT1, ADA, PYGL, P4HA1, LDHA, G6PD, NT5E, and FTH1. The concordance indices (C-indices) were 0.638 in the TCGA training set, 0.651 in the internal test set, and 0.604 in GSE65858. In a complete-case clinical cohort of 473 TCGA patients, a model combining the risk score with age, sex, and T, N, and M categories achieved C-indices of 0.693 in TCGA and 0.677 in GSE65858. The corresponding 3-year areas under the receiver operating characteristic curve (AUCs) were 0.735 and 0.700. Single-cell analysis showed that the eight genes were distributed across malignant epithelial, proliferating, immune, endothelial, and stromal populations. The 16,240 copy number variation-high (CNV-high) epithelial cells had higher eight-gene scores and higher transcriptional scores for glycogen metabolism, the pentose phosphate pathway, glycolysis, and redox-related programs. Small interfering RNA (siRNA)-mediated silencing of G6PD or PYGL reduced relative wound closure at 12 h in TU686 and SCC25 cells.

**Conclusion:**

The eight-gene metabolic score provided moderate and cohort-dependent prognostic discrimination in the TCGA internal test set and GSE65858. Its bulk transcriptomic signal arose from several cell populations. Silencing PYGL or G6PD reduced target protein expression and wound closure. These findings provide preliminary *in vitro* evidence linking both genes to a migratory phenotype in HNSCC.

## Introduction

Head and neck cancer (HNC) currently ranks as the sixth most common malignancy globally, accounting for 4.7% of all cancer cases and 4.9% of cancer-related deaths, making it the seventh leading cause of cancer mortality worldwide 1. Among these, head and neck squamous cell carcinoma (HNSCC) is the most prevalent histological subtype 2. Its treatment affects not only tumor control but also speech, swallowing, breathing, appearance, and social function. Patients with localized disease usually undergo surgery or radiotherapy. Patients with locoregionally advanced disease often receive surgery followed by radiotherapy or chemoradiotherapy, or definitive concurrent chemoradiotherapy. Recurrent or metastatic disease may be treated with immunotherapy, with or without chemotherapy. Despite advances in multidisciplinary care, long-term survival remains unsatisfactory in patients with locoregionally advanced, recurrent, or metastatic disease. The need to balance tumor control with preservation of function also creates an ongoing clinical need for individualized risk assessment 3.

HNSCC is not a single disease. It is a heterogeneous group shaped by anatomic site, carcinogenic exposure, viral infection, and molecular alterations. Tobacco and alcohol remain major risk factors. Human papillomavirus (HPV), especially high-risk HPV, is a major cause of a subset of oropharyngeal squamous cell carcinomas. HPV-positive oropharyngeal cancer differs from HPV-negative disease in epidemiology, molecular pathogenesis, treatment response, and prognosis. The eighth edition of the American Joint Committee on Cancer and Union for International Cancer Control (AJCC/UICC) staging system therefore uses separate staging systems for these tumors 4. Marked genomic and transcriptomic heterogeneity is also present within HPV-negative HNSCC. Studies including TCGA have identified complex somatic alterations, molecular subtypes, and immune features in HNSCC. However, these molecular differences are not fully captured by conventional anatomic staging 5. Patients with similar T, N, and M categories and similar treatments may therefore have very different patterns of recurrence and survival. Clinicopathologic variables alone cannot fully describe the biological risk of an individual tumor.

Metabolic reprogramming links tumor-intrinsic alterations to adaptation within the tumor microenvironment. To sustain growth, tumor cells must coordinate energy production, nucleotide and lipid synthesis, protein translation, and redox balance. They must also adjust metabolic flux according to the availability of oxygen, glucose, and other nutrients. Oncogenic drivers can alter metabolic enzyme expression and substrate use. Hypoxia, nutrient competition, and treatment pressure then select cell states that can survive under adverse conditions. Metabolic changes are not limited to malignant cells. The metabolic states of cancer-associated fibroblasts, endothelial cells, and infiltrating immune cells can affect extracellular matrix remodeling, angiogenesis, immune activity, and immunosuppression. These processes shape the tumor microenvironment 6. Metabolic signals in HNSCC may therefore reflect both the intrinsic needs of malignant epithelial cells and changes in the composition of immune and stromal populations.

Several studies have used bulk transcriptomic data to extract metabolism-related prognostic information. Du et al. developed a seven-gene metabolism-related prognostic index that included HPRT1 and ADA. They evaluated its survival stratification, metabolic features, and immune phenotype in TCGA and Gene Expression Omnibus (GEO) cohorts 7. Cao et al. reported another seven-gene metabolic risk signature that included HPRT1, PYGL, ADA, and P4HA1. The model showed some ability to predict OS in TCGA and GSE65858 8. A recent study integrated single-cell and bulk transcriptomic data. It described heterogeneity in serine, glycine, and one-carbon metabolism among malignant HNSCC cells and used these findings to develop a prognostic signature and explore potential metabolic features 9. These studies show that metabolism-related transcriptional signals can add prognostic information beyond conventional staging. They also suggest that some metabolic genes recur across different modeling frameworks.

Although metabolism-related prognostic models have been reported in many cancers, their clinical reproducibility and biological interpretation remain limited. Most published models were developed from bulk transcriptomic data. Their performance may vary with the expression platform, patient population, and survival endpoint. Independent cohort validation should therefore use a fixed risk formula and a fixed grouping cutoff 10. Bulk expression also represents a mixture of several cell types within tumor tissue. Changes measured in whole tissue cannot identify the main cellular source of a risk gene 11, 12. Pathway enrichment and immune infiltration analyses can provide biological clues, but they should be combined with single-cell localization and functional experiments to further assess the cellular phenotypes associated with key genes.

Therefore, we established a layered evidence framework that combined clinical cohorts, single-cell analysis and *in vitro* experiments. We used differential expression and survival data from TCGA-HNSC to screen metabolism-related candidate genes and develop an eight-gene risk score. We assessed OS stratification and added clinical value in the TCGA internal test set and GSE65858. GSE117973 used DSS and was included only for exploratory evaluation. At the single-cell level, we described the distribution of the eight genes across cell types. We also examined CNV-associated epithelial states, prespecified metabolic modules, the context of cell-cell communication, and computational perturbation networks related to PYGL and G6PD. We then performed preliminary *in vitro* functional experiments for PYGL and G6PD. The aim was to develop an integrated HNSCC prognostic model that combined metabolic genes with clinical factors. We also aimed to place cross-cohort prognostic evidence, single-cell heterogeneity, and preliminary functional validation within the same framework. This approach may help identify key metabolism-related differentially expressed genes for future prognostic and therapeutic research in HNSCC.

## Methods

### Selection of datasets

To screen for metabolism-related genes and construct a prognostic model, we selected datasets based on the following inclusion criteria: (1) Pathologically confirmed tumor tissues; (2) Availability of complete clinical annotations for each patient; and (3) RNA sequencing (RNA-seq), microarray profiling, or single-cell RNA sequencing (scRNA-seq) performed on fresh-frozen biopsy specimens. All experimental data utilized in this study were retrieved from publicly accessible repositories in strict adherence to data access policies and publication guidelines. Consequently, ethical review board approval or informed consent was not required.

### Study design and Acquisition of Bulk RNA-seq and Single-Cell Data

The study included four stages: bulk transcriptomic modeling, clinical model evaluation, single-cell analysis, and preliminary *in vitro* functional evaluation (Figure 1). Transcriptomic and clinical data for TCGA-HNSC were obtained from TCGA through the Genomic Data Commons portal 13. Transcriptomic and clinical data for external evaluation were obtained from GEO. GSE65858 was used as an external OS evaluation cohort. Its original study provided gene expression, HPV-related information, and survival annotations 14. GSE117973 used DSS as its endpoint and was included only in exploratory analyses 15. scRNA-seq data were obtained from the original studies of GSE150321 (n = 2) and GSE172577 (n = 6) 16, 17. Metabolism-related gene sets were obtained from the Molecular Signatures Database (MSigDB) 18.

The TCGA differential expression analysis included 515 tumor samples and 44 normal samples. Among these, 506 patients had complete expression and survival data. After stratification by outcome events, the data were divided into a training set and an internal test set at a 7:3 ratio using a random seed of 123. The training set comprised 355 patients and 132 death events, while the internal test set comprised 151 patients and 56 death events. The transcriptomic survival cohort therefore comprised 506 patients (training, n = 355; internal test, n = 151). After complete-case filtering for clinicopathological variables, 473 patients remained for the clinical analyses, including 332 from the original training subset and 141 from the original internal test subset. The GSE65858 dataset included 270 patients and 94 overall survival (OS) events, and the GSE117973 dataset included 77 patients and 16 disease-specific survival (DSS) events. To evaluate the model's sensitivity to data partitioning, we conducted 500 iterations of stratified repeated partitioning for each of three ratios, 6:4, 7:3 and 8:2, while keeping the candidate gene pool and the LASSO + StepCox workflow constant. The key clinicopathological characteristics of each cohort are presented in Tables 1-3.

### Metabolism-related differential expression and candidate gene screening

After gene annotation and preprocessing, tumor and normal samples in TCGA were compared using the limma package 19. The Benjamini-Hochberg method was used to control for multiple testing. Metabolism-related differentially expressed genes (MRDEGs) were defined using prespecified thresholds of |log fold change| > 0.5 and false discovery rate (FDR) < 0.05. Univariate Cox proportional hazards regression was performed separately for each of the 936 metabolism-related genes using only the TCGA training set (n = 355; 132 overall survival events). Genes with *p* < 0.05 were considered prognosis related genes. Their intersection with the MRDEGs formed the initial candidate gene set.

### Construction of the Prognostic Model

The 12 candidate genes shared across cohorts were HPRT1, ADA, PYGL, SMS, P4HA1, LDHA, G6PD, TXNRD1, NT5E, PTDSS1, FTH1 and CHST11. To reduce platform-related differences, each gene was independently Z-standardized within each cohort. All survival-based feature selection and model fitting procedures were performed using the TCGA training set. The multi-algorithm framework included regularized Cox regression, stepwise Cox regression (StepCox), CoxBoost, partial least squares Cox regression, random survival forests, gradient boosting, supervised principal components for survival, and survival support vector machines. In total, 126 algorithm combinations were evaluated, and 124 ran successfully. To examine potential selection bias arising from the comparison of 126 algorithmic configurations, we repeated the analysis using nested cross-validation restricted to the original TCGA training cohort (n = 355; 132 events). The outer procedure consisted of 10 repeats of stratified five-fold cross-validation. In each outer split, feature selection was rerun using only the outer-training data. Hyperparameters for penalized models were selected within the same data, whereas models without tunable hyperparameters retained their prespecified settings. For each repeat, predictions from the five held-out folds were combined to calculate the Harrell C-index for all 355 patients. The configurations were then ranked according to their mean C-index across the 10 repeats. The TCGA internal test set, GSE65858, and GSE117973 were not used for feature selection, parameter tuning, or model ranking. Within each outer-training fold, LASSO feature selection was performed using 10-fold cross-validation, with lambda.min used to select the penalty parameter. A multivariable Cox proportional hazards model was then fitted using the retained genes 20. No additional stepwise elimination was performed after LASSO selection. This combination was labeled LASSO + StepCox in the algorithm comparison plot. The final risk score was calculated as follows: Risk score = 0.1689 × HPRT1 + 0.2172 × ADA + 0.1844 × PYGL + 0.0875 × P4HA1 + 0.1187 × LDHA + 0.1401 × G6PD + 0.1266 × NT5E + 0.0854 × FTH1. Each expression value was Z-standardized within the corresponding cohort. The median risk score in the TCGA training set, -0.03298, was prespecified as the cutoff and applied to the internal test set and external cohorts. The configuration with the highest mean out-of-fold C-index in the training-only analysis was considered the best-performing model by this metric. LASSO + StepCox was selected as the primary model because its explicit coefficients and compact eight-gene structure facilitated biological interpretation. It also showed relatively consistent performance across cohorts when applied without refitting. None of the held-out cohorts was used to estimate model coefficients or tune hyperparameters. However, we considered the performance in GSE65858 when deciding which model to emphasize. GSE65858 is therefore regarded as an external assessment of model transportability rather than a fully selection-naive validation cohort.

### Survival assessment and clinical nomogram

Discrimination was evaluated using Harrell's C-index and time-dependent receiver operating characteristic (ROC) analysis 21. Prespecified high-risk and low-risk groups were compared using Kaplan-Meier curves and the log-rank test. Cox regression was used to assess the association between the continuous risk score and survival. AUCs at 1, 3, and 5 years were calculated when follow-up duration allowed. The main clinical analysis excluded treatment information and included 473 TCGA patients with complete clinicopathological data, comprising 332 patients from the original training subset and 141 from the original internal test subset. Candidate variables were the risk score, age, sex, smoking history, alcohol history, and T, N, and M categories. Composite stage was not included. Each variable was first assessed in univariate Cox analysis. A global likelihood ratio test with *p* < 0.05 was required for entry into the multivariable model. The same complete-case population was used for univariate and multivariable analyses. The final nomogram was based on the selected variables. Calibration at 1, 3, and 5 years was evaluated using 200 bootstrap resamples. External evaluation was performed in GSE65858. The median linear predictor from the TCGA model, 1.2496, was used as a fixed cutoff in GSE65858.

The extended analysis that included treatment information used 467 TCGA patients with complete treatment data. Treatment was grouped as surgery alone (S), surgery plus radiotherapy (S+RT), and surgery plus chemoradiotherapy (S+CRT). Surgery alone was the reference category. The same univariate screening criteria and complete-case strategy used in the main model were applied. The model was evaluated in GSE117973 only as an exploratory DSS analysis. Available treatment variables were recorded in broad categories, and the exploratory GSE117973 cohort was small. Treatment coefficients were therefore interpreted only as observational prognostic associations and were not used to infer treatment benefit. The proportional hazards assumption was assessed using Schoenfeld residuals.

### Metabolic pathway analysis

To characterize the biological processes associated with the fixed eight gene risk score, we evaluated 10 prespecified pathways, including glycogen metabolism, the pentose phosphate pathway (PPP), glycolysis, oxidative phosphorylation, reactive oxygen species (ROS) response, glutathione metabolism, ferroptosis-related processes, hypoxia, purine metabolism, and extracellular matrix (ECM) remodeling. Pathway scores were calculated using ssGSEA within each bulk cohort and Z-standardized within cohorts. Spearman correlation was used to assess the relationships between pathway scores and the fixed risk score, and univariable Cox regression was used to assess their associations with survival. In addition, GSEA based on the complete transcriptome was performed between the prespecified high- and low-risk groups.

### Single-cell quality control, integration, and cell annotation

Single-cell analyses used random seed 42. Only singlets with nFeature RNA > 200 and < 7,000, nCount RNA < 50,000, and percent.mt < 20% were retained. Data were normalized using the LogNormalize method in Seurat with a scale factor of 10,000. The 2,000 most variable genes were selected, and principal component analysis (PCA) was performed 22. Samples were then integrated using Harmony 23. A neighbor graph was constructed using the first 20 dimensions. Clustering at a resolution of 0.3 identified 19 clusters among 54,163 cells. Cell identities were assigned using canonical lineage markers, cluster-specific differentially expressed genes, cell-type signature scores, and annotations from the original studies. The cells were grouped into 14 populations: Malignant-epithelial, Cycling malignant cell, Goblet like secretory epithelial cell, Secretory salivary epithelial cell, CD8 cytotoxic T cell, CD4 T cell, Cycling T cell, B cell, Plasma cell, Myeloid macrophage, Fibroblast CAF, Pericyte vSMC, Endothelial, and Mast cell.

### InferCNV and identification of malignant epithelial cells

InferCNV was run separately for each sample. Large-scale copy number variation (CNV) patterns were estimated from chromosomal patterns in single-cell transcriptomic expression. This was an RNA-based computational inference 24, 25. The four epithelial populations were treated as potentially malignant cells. B cells, plasma cells, myeloid cells and macrophages, fibroblasts, endothelial cells, and pericytes and vascular smooth muscle cells were used as reference cells. Each sample was required to include at least 20 potentially malignant cells and 30 reference cells. The parameters were cutoff = 0.1, cluster-by-groups = TRUE, denoise = TRUE, hidden Markov model (HMM) = FALSE, analysis-mode = "subclusters", and num-threads = 4. The CNV score for each cell was defined as the sum of squared differences between the inferCNV-smoothed expression values and 1. The within-sample threshold was the larger of the 95th percentile of the reference-cell scores and the reference-cell mean plus two standard deviations. Potentially malignant cells above this threshold were classified as CNV-high malignant cells. The remaining cells were classified as CNV-low epithelial cells. This definition does not represent CNV confirmed by DNA sequencing.

### Prespecified metabolic module scoring

In the single-cell analysis, the eight-gene score and the G6PD/PYGL anchor were added to the 10 pathways described above, resulting in 12 prespecified modules. For each module, module scores were calculated as the mean log-normalized expression of the matched genes. Mean module scores across cell populations were Z-standardized. CNV-high and CNV-low epithelial cells were compared using the Wilcoxon rank-sum test with Benjamini-Hochberg correction.

### CellChat, within-sample target-high and target-low states, and computational perturbation analysis

CellChat version 1.6.1 was applied to normalized expression data from the 14 cell types. Candidate cell-cell communication networks were inferred within a ligand-receptor database framework 26. We focused on pathways related to MIF, SPP1, CXCL, CCL, VEGF, collagen, laminin, FN1, and THBS.

PYGL and G6PD expression was standardized within each sample among CNV-high Malignant epithelial and Cycling malignant cells. The lowest and highest quartiles defined target-low and target-high cells. Samples with fewer than 30 eligible cells were not grouped. If the two quartile thresholds were identical, the 10th and 90th percentiles were used instead. The dual score was the mean of the within-sample PYGL and G6PD Z scores. Module scores and selected communication-related ligand genes were compared with the Wilcoxon rank-sum test, followed by Benjamini-Hochberg correction. This analysis compared naturally occurring expression states. It was not an experimental gene knockdown.

A total of 3,000 cells were sampled from CNV-high Malignant epithelial and Cycling malignant cells. These included 2,637 Malignant epithelial cells and 363 Cycling malignant cells. Raw counts for 3,021 filtered genes were analyzed with scTenifoldKnk to generate candidate network-perturbation predictions after virtual knockout of PYGL or G6PD 27. Genes with adjusted *p* < 0.05 were considered computationally significant network-perturbation candidates. Gene Ontology (GO) enrichment for biological process (BP), cellular component (CC), and molecular function (MF) was performed with clusterProfiler and org.Hs.eg.db 28. The *p*-value cutoff was 0.05, and the q-value cutoff was 0.25.

### Cell culture and siRNA transfection

The human tongue squamous cell carcinoma cell line SCC25 was provided by Professor Junbo Tu from the College of Stomatology, Xi'an Jiaotong University. The human laryngeal cancer cell line TU686 was obtained from the Second Affiliated Hospital of Xi'an Jiaotong University. Cells were cultured in Roswell Park Memorial Institute 1640 medium (RPMI-1640; Gibco) supplemented with 10% fetal bovine serum (FBS; Gibco), 100 U/mL penicillin, and 100 μg/mL streptomycin (Gibco) at 37°C in 5% carbon dioxide. Specific siRNAs targeting PYGL or G6PD (siPYGL and siG6PD) and a negative control siRNA (siNC) were supplied by GenePharma (Shanghai, China). Transfection was performed with Lipofectamine 8000 (Beyotime, Shanghai, China) according to the manufacturer's instructions. The stock concentration of each siRNA duplex was 20 μM. The oligonucleotide sequences used in the reported experiments were as follows (sense and antisense strands are shown 5'-3'): siPYGL (PYGL-Homo-1326), sense 5'-GGCCUUGUUUCCUAAAGAUTT-3' and antisense 5'-AUCUUUAGGAAACAAGGCCTT-3'; siG6PD (G6PD-Homo-510), sense 5'-GGCCGUCACCAAGAACAUUTT-3' and antisense 5'-AAUGUUCUUGGUGACGGCCTT-3'; and siNC, sense 5'-UUCUCCGAACGUGUCACGUTT-3' and antisense 5'-ACGUGACACGUUCGGAGAATT-3'.

### Western blotting

Total protein was collected from SCC25 and TU686 cells 48 h after transfection. Proteins were separated by 10% sodium dodecyl sulfate-polyacrylamide gel electrophoresis (SDS-PAGE) and transferred to a 0.2 μm polyvinylidene fluoride (PVDF) membrane at a constant current of 300 mA for 60 min. The membranes were blocked for 1 h at room temperature in buffer containing 0.1% Tween-20 and 5% nonfat milk. Primary antibodies were incubated overnight at 4 °C. After washing, the membranes were incubated with the corresponding secondary antibodies for 1 h at room temperature. Signals were detected using enhanced chemiluminescence (ECL). A mouse monoclonal antibody against β-actin (Cat. No. 66009-1-Ig, Proteintech) was used as the loading control. Primary antibodies against PYGL (Cat. No. 15851-1-AP, Proteintech) and G6PD (Cat. No. 25413-1-AP, Proteintech) were both diluted 1:1,000.

Band intensity was normalized to β-actin and then to the corresponding siNC group, with siNC set to 1. Groups were compared using a two-sided paired t test.

### Wound-healing assay

SCC25 and TU686 cells were seeded in six-well plates and transfected with the indicated siRNA or siNC. At approximately 90% confluence, a scratch was created with a 200 μL pipette tip. Cells were washed with phosphate-buffered saline (PBS) and imaged at 0 h. They were then cultured for 12 h in medium containing 2% FBS and imaged again in the same field. The wound area at 0 and 12 h was measured using ImageJ. Relative wound closure was calculated for each well and normalized to the siNC group. A one-way analysis of variance (ANOVA) was performed within each cell line. Dunnett's multiple-comparison test was used to compare siG6PD and siPYGL with siNC.

### Statistical analysis

All tests were two-sided unless stated otherwise. Multiple comparisons were corrected using the Benjamini-Hochberg or Dunnett method. Continuous variables are presented as the mean ± standard deviation unless otherwise stated in the figure. Transcriptomic and single-cell analyses were performed in R version 4.4.1. Statistical analyses for the cell experiments were obtained from GraphPad Prism 11 project files. *p* < 0.05 was considered statistically significant.

## Results

### Initial screening of metabolism-related differentially expressed genes

To identify metabolism-related genes with altered expression in HNSCC, we obtained metabolism-related gene sets from MSigDB and compared the transcriptomic profiles of 515 tumor samples and 44 normal samples in TCGA. Using thresholds of |log fold change| > 0.5 and FDR < 0.05, we identified 485 MRDEGs. Of these, 291 were upregulated and 194 were downregulated (Figure 2; Table S1). In the TCGA training set (n = 355; 132 overall survival events), univariate Cox analysis of 936 metabolism-related genes identified 108 genes associated with OS at *p* < 0.05. The intersection of these 108 prognosis-related genes and the 485 MRDEGs yielded 59 initial candidate genes, including 35 upregulated and 24 downregulated genes.

### The eight-gene score stratified survival in TCGA and GSE65858

Repeated nested cross-validation in the TCGA training set ranked survival-SVM first (mean out-of-fold C-index, 0.625) and LASSO + StepCox 30th (0.601). In the internal test set and GSE65858, the corresponding C-indices were 0.651 and 0.604 for LASSO + StepCox, versus 0.675 and 0.593 for survival-SVM. We retained LASSO + StepCox because its explicit coefficients, compact gene set, and consistent cross-cohort performance supported interpretability and transportability. As GSE65858 informed this decision, it was treated as an external transportability assessment rather than a fully selection-naive validation cohort. The final model included HPRT1, ADA, PYGL, P4HA1, LDHA, G6PD, NT5E, and FTH1, with all coefficients being positive (training C-index, 0.638; Figure 3A; Table S2).

When the training-set median of -0.03298 was used as the cutoff, the high-risk group had poorer survival in all three OS cohorts. Expression heatmaps also showed that the model genes tended to have higher expression in the high-risk groups. This pattern was consistent with stratification by the risk score (Figure S2). In the TCGA training set, the low-risk and high-risk groups included 177 and 178 patients, with 45 and 87 deaths, respectively. Median OS was 2,703 days in the low-risk group and 882 days in the high-risk group. The hazard ratio (HR) was 2.535(95% CI 1.756-3.660, log-rank *p* = 2.87 x 10^-7^; Figure 3B). In the internal test set, the low-risk and high-risk groups included 75 and 76 patients, with 22 and 34 deaths. Median OS was 1,972 and 1,079 days, respectively. The HR was 1.781 (95% CI 1.039-3.054; log-rank *p* = 0.0332; Figure 3C). In GSE65858, the low-risk and high-risk groups included 128 and 142 patients, with 39 and 55 deaths. Median OS was 1,939 and 1,214 days, respectively. The HR was 1.718 (95% CI 1.127-2.619; log-rank *p* = 0.0112; Figure 3D).

The HRs for the continuous risk score were 2.718 in the TCGA training set (*p* = 2.11 x 10^-9^), 1.914 in the internal test set (*p* = 1.05 x 10^-4^), and 1.658 in GSE65858 (*p* = 0.00512). The 1-, 3-, and 5-year AUCs were 0.628, 0.707, and 0.668 in the training set; 0.709, 0.666, and 0.533 in the internal test set; and 0.674, 0.652, and 0.731 in GSE65858 (Figure 3E-G). GSE117973 used DSS as the endpoint, and the score was not validated in this cohort. The C-index was 0.436, and the HR was 0.807 (95% CI 0.323-2.013; *p* = 0.645). The 1-, 2-, and 3-year AUCs were 0.319, 0.392, and 0.380 (Figure S3).

Sensitivity analyses showed similar overall discrimination across different training-to-test split ratios. HPRT1, ADA, and PYGL were retained in more than 91% of repeated analyses, whereas the remaining genes showed some sampling variability. However, coefficient directions remained highly consistent when the genes were selected. Overall model performance was therefore relatively insensitive to the data split, and the original 7:3 ratio provided a reasonable balance between model fitting and internal evaluation (Figure S4).

### Development and validation of the clinical nomogram

To assess the clinical prognostic value of the eight-gene risk score and its complementarity with conventional clinical indicators, we summarized the clinicopathological characteristics of the TCGA training set, TCGA internal test set, GSE65858, and GSE117973 (Table 1-3).

The cross-cohort model without treatment information included 473 TCGA patients with complete clinical data, of whom 174 died. After univariable screening at P < 0.05, the risk score, age, sex, and T, N, and M categories entered the multivariable model. Smoking and alcohol history did not meet the prespecified criterion. In univariable analysis, a higher risk score was associated with poorer OS (HR = 2.228, 95% CI 1.787-2.779; *p* = 1.18 x 10^-12^). The association remained after adjustment for the other selected variables (HR = 2.162, 95% CI 1.736-2.692; *p* = 5.65 x 10^-12^). N category (HR = 1.462, 95% CI 1.227-1.743; *p* = 2.23 x 10^-5^) and M1 category (HR = 2.889, 95% CI 1.035-8.066; *p* = 0.043) also remained significant (Figure 4A, B). The multivariable model showed no clear violation of the proportional hazards assumption (global *p* = 0.968; Table S3).

Based on the variables that met the prespecified univariable screening criterion, we constructed a nomogram incorporating the risk score, age, sex, and T, N, and M categories to estimate 1-, 3-, and 5-year overall survival (Figure 4C). The model achieved C-indices of 0.693 in TCGA and 0.677 in the GSE65858 external evaluation cohort (Table S4). The time-dependent AUCs at 1, 3, and 5 years were 0.704, 0.735, and 0.654 in TCGA and 0.746, 0.700, and 0.740 in GSE65858, respectively (Figure 4D, E). At 3 years, the complete nomogram showed greater discrimination than the risk score or any individual clinical predictor, with AUCs of 0.735 in TCGA and 0.700 in GSE65858 (Figure 4F, G). Using the median TCGA linear predictor of 1.2496 as a fixed cutoff, the model significantly stratified overall survival in TCGA and GSE65858, with log-rank P values of 2.01 × 10^-11^ and 7.28 × 10^-5^, respectively. Internal calibration was performed using 200 bootstrap resamples, followed by external calibration in GSE65858 (Figure S5; Table S5).

In the treatment-extended analysis, 467 TCGA patients with complete data and 171 deaths were included. The risk score, age, T category, N category, and treatment met the prespecified univariable screening criterion and entered the multivariable model (Figure S6A, B). The risk score remained independently associated with poorer overall survival (HR = 2.29, 95% CI 1.82-2.89; *p* < 0.001), whereas N category was also independently associated with prognosis (HR = 1.59, 95% CI 1.32-1.91; *p* < 0.001). Relative to surgery alone, surgery plus radiotherapy and surgery plus chemoradiotherapy were associated with lower observed hazards of death, with HRs of 0.56 (95% CI 0.38-0.81; *p* = 0.002) and 0.45 (95% CI 0.30-0.67; *p* < 0.001), respectively. A treatment-extended nomogram incorporating the risk score, age, T category, N category, and treatment achieved a C-index of 0.698 and a 3-year AUC of 0.729 in TCGA (Figure S6C, F; Table S4). Using the fixed TCGA cutoff of 0.9644, significant survival stratification was observed in TCGA (log-rank P = 3.86 × 10^-12^), but not in GSE117973 (log-rank P = 0.587; Figure S6D, E; Table S5). In GSE117973, the model yielded a C-index of 0.547 and a 3-year disease-specific survival AUC of 0.523 (Figure S6G; Table S4). Given the small number of disease-specific deaths and the different survival endpoint, the GSE117973 analysis was considered exploratory.

### Functional composition of the eight genes and selection of PYGL and G6PD as candidate anchors

The eight genes represented several metabolic and microenvironmental processes. These included PYGL related glycogen breakdown, G6PD related PPP activity, LDHA related glycolysis, HPRT1, ADA and NT5E related purine or adenosine metabolism, FTH1 related iron homeostasis, and P4HA1 related collagen modification and ECM remodeling (Table S6). Previous studies have reported roles for PYGL, G6PD, HPRT1, P4HA1, LDHA, NT5E, and FTH1 in HNSCC or related squamous cancer phenotypes 29-41.

Pathway level analysis further showed that glycolysis, hypoxia, purine metabolism, and collagen and ECM remodeling were generally positively associated with the fixed eight gene risk score across the main cohorts, although their associations with survival varied across datasets. GSEA based on the complete transcriptome also identified differences in metabolic, redox related, and extracellular matrix programs between the high and low risk groups, but not all pathways showed consistent support across cohorts (Figure S7A-C).

Based on their metabolic functions and expression in malignant epithelial cells, PYGL and G6PD were selected as candidate metabolism genes for further analysis. We did not assume that they formed a proven linear metabolic axis.

### Heterogeneous distribution of the eight genes across malignant, immune, and stromal cells

To define the distribution of the key MRDEGs identified in HNSCC, we extended the analysis to scRNA-seq data. After quality control and doublet removal, uniform manifold approximation and projection (UMAP) was used for dimensionality reduction and clustering. A total of 54,163 cells were assigned to 14 populations, including 26,620 Malignant epithelial cells and 3,379 Cycling malignant cells (Figure 5A). UMAP feature plots, dot plots, and mean-expression heatmaps for the eight genes showed that HPRT1 was higher in Cycling T and Cycling malignant cells. ADA was higher in Cycling malignant, Cycling T, and Endothelial cells. PYGL, LDHA, and G6PD were mainly enriched in Cycling malignant and Malignant epithelial cells. P4HA1 was highest in Fibroblast CAF cells and remained expressed in malignant epithelial and myeloid or macrophage populations. NT5E was mainly detected in Fibroblast CAF and Endothelial cells. FTH1 was higher in Myeloid macrophage cell, Mast cell, and Fibroblast CAF populations (Figure 5B-D). This pattern was consistent with the heterogeneity of malignant, immune, and stromal populations reported in previous single-cell studies of HNSC 11, 12.

To better identify malignant tumor cells, inferCNV was applied separately to each sample 42. The analysis included 34,386 potentially malignant epithelial cells and 9,751 reference cells. Of the epithelial cells, 16,240 were classified as CNV-high and 18,146 as CNV-low. CNV-high cells accounted for 55.8% of the Cycling malignant population and 51.0% of the Malignant epithelial cells. The corresponding proportions were 17.5% in Goblet like secretory epithelial cells and 17.4% in Secretory salivary epithelial cells (Figure S8). These differences supported the malignant identity of the Malignant epithelial and Cycling malignant cells. They also indicated the presence of CNV-low epithelial regions.

### CNV-high epithelial cells showed selective metabolic and redox activation

Cycling malignant and Malignant epithelial cells had high standardized scores for the G6PD/PYGL anchor, PPP, glycolysis, ROS response, glutathione metabolism, and hypoxia modules. In Cycling malignant cells, the Z scores for the anchor, PPP, glycolysis, ROS response, glutathione metabolism, and purine metabolism were 2.30, 2.10, 2.00, 1.87, 1.76, and 2.26, respectively. In Malignant epithelial cells, the Z scores for hypoxia, PPP, glycolysis, glutathione metabolism, and ROS response were 1.99, 1.75, 1.65, 1.53, and 1.42 (Figure 6A).

Compared with the 18,146 CNV-low epithelial cells, the 16,240 CNV-high cells had higher scores for the eight-gene signature (0.948 vs 0.581), G6PD/PYGL anchor (0.478 vs 0.173), glycogen metabolism (0.151 vs 0.105), PPP (0.530 vs 0.407), glycolysis (1.336 vs 0.929), ROS response (0.838 vs 0.757), and glutathione metabolism (0.751 vs 0.542). All adjusted *p* < 0.001. The oxidative phosphorylation score was slightly lower in CNV-high cells (0.415 vs 0.439). This finding indicated that not all metabolic programs were uniformly increased in the CNV-high state (Figure 6B; Table S7).

### Malignant epithelial cells were embedded within immune and stromal communication networks

To characterize communication between malignant epithelial cells and other components of the tumor microenvironment, CellChat analysis was performed across the 14 cell populations. A total of 67 signaling pathways related to cell-cell communication were detected in the HNSCC tumor microenvironment (Figure 7A; Table S8). The overall communication pattern showed that Malignant epithelial and Cycling malignant cells received signals from immune and stromal populations and also sent signals to other cell types. These findings indicate that the malignant epithelial compartment is an important part of the tumor microenvironment communication network.

When Malignant epithelial and Cycling malignant cells were treated as signal senders, MIF and laminin signaling were prominent. MIF-related interactions mainly involved the MIF-CD74/CD44, MIF-CD74/CXCR4, and MIF-ACKR3 axes (Figure S9A, B; Table S9). Signals from malignant epithelial cells also included VEGF signaling toward endothelial cells and a potential CXCL8-ACKR1 interaction between Cycling malignant and endothelial cells (Figure S9C, D; Table S9). CXCL1-, CXCL2-, and CXCL3-related signals mainly originated from Goblet like secretory epithelial and Secretory salivary epithelial cells rather than Malignant epithelial cells (Figure S9C). In the opposite direction, collagen, FN1, laminin, THBS, and SPP1 signals from immune and stromal cells toward Malignant epithelial and Cycling malignant cells were prominent (Figure S9B, E-H; Table S9). Fibroblast CAF cells showed strong signal output through interactions related to collagen, fibronectin, laminin, and thrombospondin. SPP1 signals mainly originated from Myeloid macrophage cells. These findings show that malignant epithelial cells do not function in isolation. They are embedded within a complex network shaped by immune cells, fibroblasts, vascular cells, and ECM signals (Figure 7A; Figure S9A-H).

The CellChat analysis was not stratified by high or low PYGL or G6PD expression. It assessed ligand-receptor interactions between malignant epithelial cells and immune or stromal populations according to cell type. These results therefore cannot be interpreted as evidence that PYGL or G6PD directly mediates cell-cell communication. Both G6PD and PYGL are intracellular metabolic enzymes, and neither appeared in the CellChat ligand-receptor results. Together with the metabolic module findings, the PYGL/G6PD-associated state is more likely to represent metabolic and redox adaptation within malignant epithelial cells. These cells were simultaneously located within tumor microenvironment networks involving MIF, SPP1, CXCL, VEGF, and ECM-related signaling.

### Target-low states were associated with lower scores across several metabolic modules

To further assess the relationship between PYGL and G6PD expression states and metabolism in malignant epithelial cells, cells were grouped within each sample according to PYGL, G6PD, or combined expression. The final groups included 3,647 PYGL-high and 8,285 PYGL-low cells, 3,704 G6PD-high and 7,238 G6PD-low cells, and 4,063 dual-high and 5,734 dual-low cells (Table S10). In all three comparisons, target-high cells had higher G6PD/PYGL anchor and eight-gene signature scores. The PYGL-low, G6PD-low, and dual-low states showed varying reductions in glycogen metabolism, PPP, glycolysis, ROS response, glutathione metabolism, hypoxia, oxidative phosphorylation, purine metabolism, ferroptosis-related processes, and collagen or ECM remodeling. All differences were statistically significant. Differences in the anchor, eight-gene signature, PPP, glycolysis, ROS response, and glutathione metabolism were especially clear between dual-high and dual-low cells (Figure 7B). After excluding PYGL and/or G6PD from the corresponding scores, sensitivity analyses showed that the overall patterns were largely preserved, suggesting that the observed differences were not solely driven by the direct inclusion of these grouping genes in the scoring formulas (Figure S7D).

We also compared selected CellChat-related ligand genes between target-high and target-low states. MIF expression was higher in PYGL-high, G6PD-high, and dual-high cells than in their corresponding low groups. Laminin-related genes, including LAMA3, LAMB3, and LAMC2, were also more highly expressed in most high-expression groups. In contrast, the directions of some CXCL- and collagen-related genes were not consistent across comparisons (Figure 7C). These findings show that high-expression states occur together with selected metabolic, immunoregulatory, and epithelial-ECM expression patterns. They should not be interpreted as the effects of gene knockdown.

### Virtual PYGL and G6PD knockout identified candidate epithelial network changes

At adjusted *p* < 0.05, scTenifoldKnk identified 49 candidate genes after virtual G6PD knockout and 44 after virtual PYGL knockout in malignant epithelial cells. Six genes were shared between the two analyses: RPS4Y1, LYAR, SFN, KRT16, KRT6C, and KRT6B (Figure 7D; Table S11). The adjusted *p* values after PYGL and G6PD knockout were 0.0018 and 0.0355 for SFN, 0.0035 and 0.0099 for KRT16, and 0.0080 and 0.0120 for KRT6B, respectively. CLDN7 was significant only after G6PD knockout (adjusted *p* = 8.23 × 10⁻¹² vs 1.00 after PYGL knockout). Enrichment analysis linked G6PD-related genes mainly to epithelial junctions, membrane regions, and intermediate filaments, whereas PYGL-related genes were associated with keratinization, epidermal differentiation, and intermediate filament organization (Table S12).

### Silencing PYGL or G6PD reduced target protein expression and wound closure at 12 h

To test whether the PYGL- and G6PD-related signals prioritized in the computational analyses were associated with HNSCC cell phenotypes, TU686 and SCC25 cells were transfected with siG6PD, siPYGL or siNC. Western blotting confirmed reduced expression of the corresponding target protein in both cell lines (Figure 8A, B). Wound-healing assays showed that silencing either PYGL or G6PD reduced wound closure in both cell lines (Figure 8C, D).

## Discussion

This study integrated several bulk transcriptomic cohorts with two scRNA-seq datasets. We developed and interpreted an HNSCC prognostic score based on HPRT1, ADA, PYGL, P4HA1, LDHA, G6PD, NT5E, and FTH1. We then examined the score using single-cell data and preliminary *in vitro* experiments. The score was associated with OS in the TCGA training set, internal test set, and GSE65858. After adjustment for age, sex, and tumor, node, and metastasis (TNM) categories, the risk score retained independent prognostic information. A nomogram that included clinical variables showed some improvement over the risk score alone. The score was not validated in GSE117973, which used DSS as its endpoint. The main finding was not a proven single metabolic pathway. Instead, the score represented a composite transcriptional signal with cross-cohort prognostic information. This signal included malignant epithelial, proliferating, immune, and stromal components. The continuous risk score was associated with poorer survival in all three OS cohorts. The C-indices were 0.638 in the TCGA training set, 0.651 in the internal test set, and 0.604 in GSE65858. The combined clinical model achieved C-indices of 0.693 in TCGA and 0.677 in GSE65858. The 3-year AUCs were 0.735 and 0.700. These values were higher than those for the risk score alone in the corresponding cohorts. The findings indicate that the risk score provides information beyond standard clinicopathologic factors. However, overall discrimination remained moderate, and the 5-year AUC in the internal test set was limited (0.533). This attenuation may reflect that baseline transcriptomic profiles capture the tumor state only at sampling, whereas subsequent treatment, recurrence, salvage therapy, and clonal evolution increasingly shape longer-term survival. The smaller number of patients remaining under observation at later time points may also increase uncertainty in the 5-year estimate. The score should therefore be interpreted as providing stronger short- to intermediate-term than long-term prognostic information. Use of the fixed TCGA median linear predictor of 1.2496 produced significant survival stratification in both cohorts. The proportional hazards tests showed no clear violation. The risk score and standard clinical variables therefore provided complementary prognostic information. The similar performance across alternative training and test splits suggests that overall model discrimination was not strongly dependent on the original 7:3 division, although individual gene selection showed some sampling variability. These results are not sufficient to support direct clinical use, and independent prospective validation is still needed.

The treatment model further illustrated the value and limitations of observational treatment information. In the internal TCGA analysis, the risk score, age, T category, N category, and treatment entered the multivariable model. The model achieved a C-index of 0.698 and a 3-year AUC of 0.729. Compared with surgery alone, surgery plus radiotherapy or chemoradiotherapy was associated with a lower observed risk of death. The negative findings in GSE117973 may reflect the change from OS to DSS, the small sample size, and the limited number of 16 events. In GSE117973, the C-index was only 0.547 and the 3-year AUC was 0.523. Neither the fixed linear predictor nor the prespecified cutoff produced significant DSS stratification. The treatment model should therefore be viewed as an exploratory internal analysis rather than a validated prognostic model.

The eight genes represented glycogen breakdown, the PPP, glycolysis, purine or adenosine metabolism, iron homeostasis, and ECM remodeling. Pathway level analyses further supported associations of the risk score with several metabolic, redox related, and extracellular matrix programs, although the strength of these associations varied across cohorts. Previous studies suggest that PYGL mediated glycogen mobilization may support tumor cell proliferation and stress adaptation 29, 30. G6PD contributes to nicotinamide adenine dinucleotide phosphate (NADPH) production and redox balance through the oxidative PPP 31, 32. P4HA1 and NT5E are linked to collagen modification and adenosine-related microenvironmental processes, respectively 35, 36, 38, 39. These known functions provide biological context. However, the positive coefficient for every gene does not mean that each gene has an independent prognostic effect of equal strength. The Wald tests for several individual coefficients were not statistically significant. The model should therefore be interpreted as a combined risk score. We focused on PYGL and G6PD because they represented two metabolism related processes of interest and were both enriched in malignant epithelial populations.

Single-cell analysis clarified the cellular sources of the bulk risk score. PYGL, LDHA, and G6PD were more highly expressed in Malignant epithelial and Cycling malignant cells. HPRT1 and ADA were enriched in Cycling malignant cells. P4HA1 was highest in Fibroblast CAF cells. NT5E was mainly detected in Fibroblast CAF and Endothelial cells. FTH1 was higher in Myeloid macrophage, Mast cell, and Fibroblast CAF populations. These findings provide a cell-composition-based explanation for the bulk risk score. They suggest that the score reflects a composite prognostic signal shaped by both the intrinsic metabolic state of malignant epithelial cells and the tumor microenvironment. Sample-specific inferCNV analysis showed high proportions of CNV-high cells in the Malignant epithelial and Cycling malignant populations. This result provided transcriptomic support for their malignant identity. A high-risk score may therefore reflect both the metabolic and proliferative state of malignant epithelial cells and the composition of the tumor microenvironment. CNV-high epithelial cells had higher scores for the eight-gene signature, glycogen metabolism, PPP, glycolysis, ROS response, and glutathione metabolism. Their oxidative phosphorylation score was slightly lower. This pattern is more consistent with selective metabolic and redox adaptation than with a uniform increase in all metabolic activity.

Within this composite signature, PYGL and G6PD were metabolic nodes of particular interest. Previous work has shown that PYGL supports glycogen mobilization, tumor cell proliferation, and stress adaptation 29, 30. G6PD maintains NADPH production and redox balance through the PPP and can promote malignant progression in HNSCC 31, 32. We found that PYGL/G6PD-high states were mainly located in CNV-high malignant epithelial cells. These states were associated with higher scores for the eight-gene signature, PPP, glycolysis, ROS response, and glutathione metabolism. These patterns remained after PYGL and/or G6PD were removed from the corresponding scores, indicating that the associations were not simply caused by including the target genes in score calculation. scTenifoldKnk identified candidate network perturbations involving squamous differentiation, keratinization, epithelial junctions, and membrane organization. In TU686 and SCC25 cells, silencing either PYGL or G6PD reduced the corresponding protein level and wound closure at 12 h. Together, these findings define a testable model of metabolic and redox adaptation involving PYGL and G6PD in HNSCC. They do not establish that PYGL-mediated glycogen breakdown directly supplies carbon to the G6PD-dependent PPP or that the two genes form a linear regulatory pathway.

The three single-cell analyses addressed different aspects of this model. CellChat characterized the communication environment surrounding malignant epithelial cells, including MIF, laminin, SPP1, CXCL, VEGF, and ECM networks 11, 12, 26. PYGL and G6PD were not ligands or receptors in these networks. Target-high and target-low comparisons captured associations with natural expression states rather than responses to gene silencing. scTenifoldKnk predicted network perturbations after virtual knockout, but the predicted changes involving SFN, KRT16, KRT6B, CLDN7, and other candidates were not experimentally examined following PYGL or G6PD silencing. Together, these analyses provide complementary context and generate specific hypotheses for further mechanistic studies. Overall, the model appears to represent a composite prognostic signal shaped by the metabolic state of malignant epithelial cells and the tumor microenvironment.

This study also has several limitations. First, it was a retrospective analysis of public data. Differences in platforms, patient populations, and endpoint definitions may have introduced heterogeneity across cohorts. GSE117973 used DSS, included only 77 patients, and had only 16 events. It cannot be considered an equivalent external validation cohort for an OS model. GSE65858 provided external evaluation, but the current model showed only moderate discrimination. Prospective cohorts with consistently collected treatment details, comorbidities, and subsequent therapy are still lacking. Second, the single-cell analysis included eight samples and cannot replace independent replication at the patient level. Cell annotation, module scoring, and CellChat depend on the selected gene sets, parameters, and databases. inferCNV also depends on the reference cells and study-specific thresholds and cannot replace DNA-based copy number measurement 42. Target-high and target-low comparisons were based on naturally occurring expression states. scTenifoldKnk provided computational predictions at the network level. Neither analysis can establish causality. Finally, although gene-silencing experiments were performed, the study lacked an independent institutional cohort, spatial validation, metabolic flux measurements, broader gene perturbation experiments, organoid models, and animal studies. The clinical value of the score and the mechanisms related to PYGL and G6PD require further investigation.

## Conclusion

We developed and evaluated an eight-gene metabolism-related risk score for HNSCC across several cohorts. The score was associated with OS in the TCGA internal test set and GSE65858. Adding clinical variables produced a limited but measurable improvement in discrimination. Single-cell analysis showed that the bulk eight-gene signal arose from malignant epithelial, proliferating, immune, endothelial, and stromal populations. CNV-high epithelial cells showed selective transcriptional features related to glycogen metabolism, PPP activity, glycolysis, and redox processes. Silencing PYGL or G6PD reduced target protein expression and wound closure at 12 h in two HNSCC cell lines. These findings provide preliminary *in vitro* evidence that links the candidate genes to a migration-related phenotype. The clinical utility of the score and the metabolic mechanisms associated with PYGL and G6PD require validation in prospective cohorts and rigorous functional studies.

## Supplementary Material

Supplementary figures.

Supplementary tables.

## Figures and Tables

**Figure 1:**
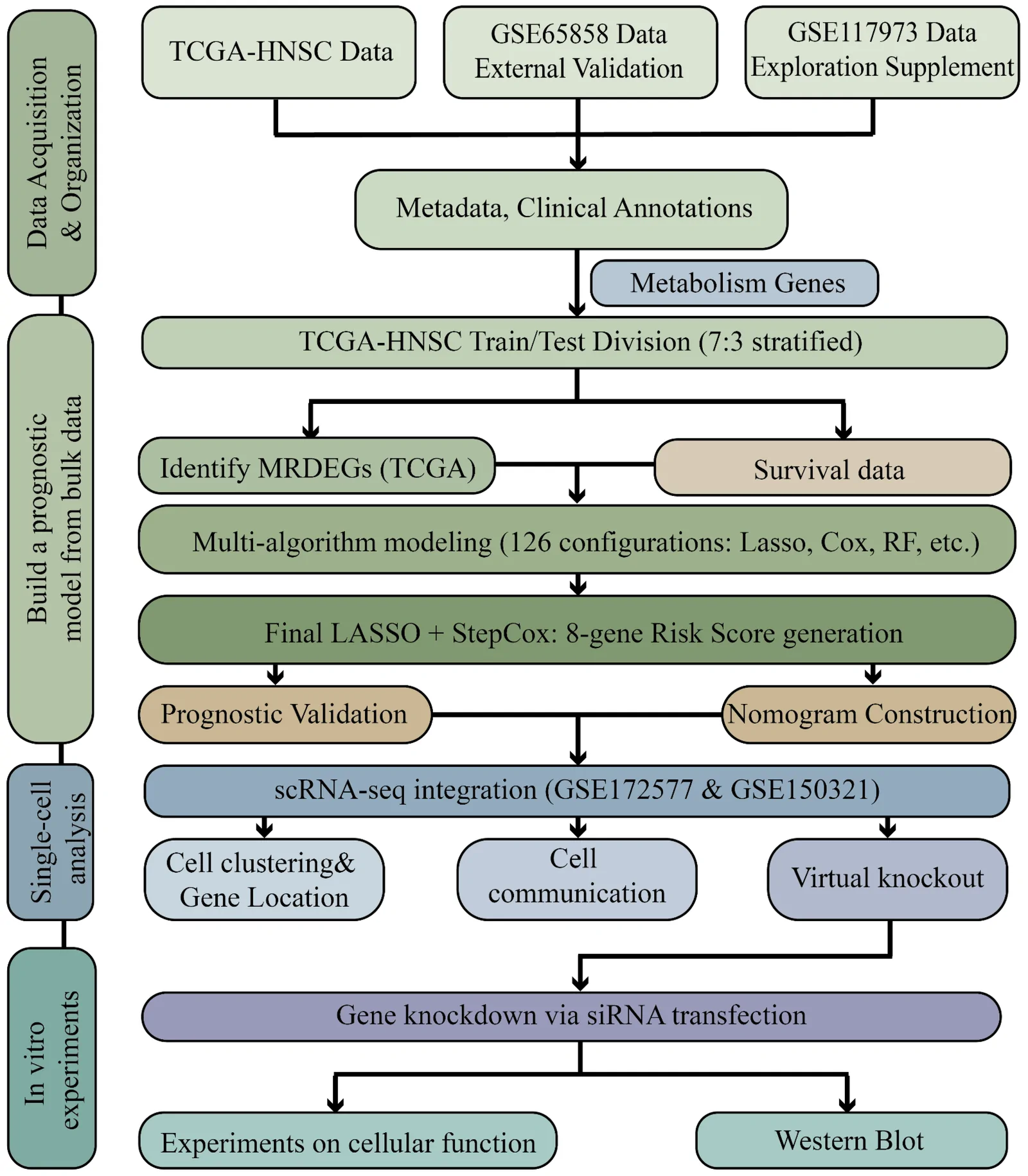
** Study design.** Overall workflow for identifying metabolism-related differentially expressed genes in TCGA, screening candidate genes, developing the cross-cohort risk model and clinical nomogram, resolving cellular sources, performing computational perturbation analysis, and conducting cell-based validation

**Figure 2:**
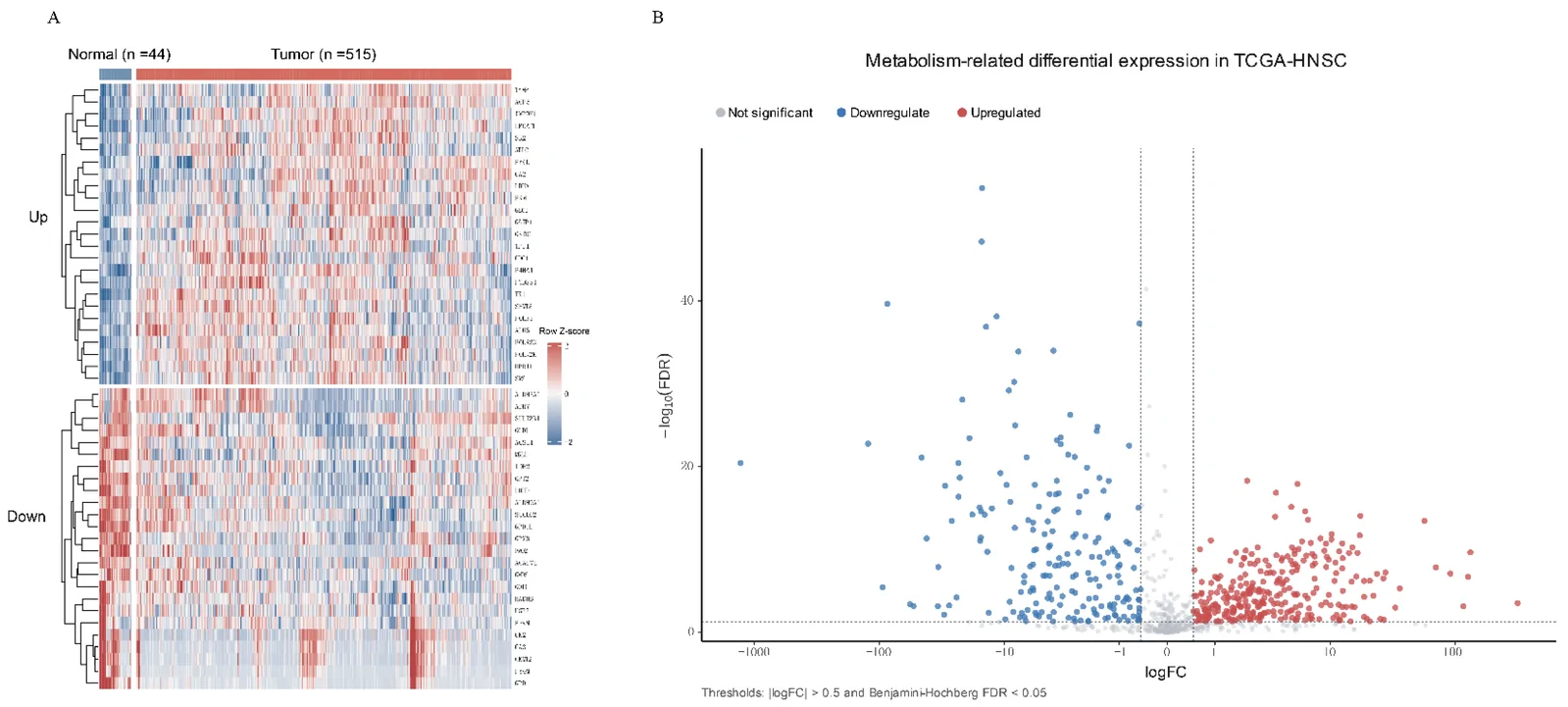
** Metabolism-related differentially expressed genes.** (A) Heatmap of 50 representative genes (25 upregulated and 25 downregulated), selected by combined effect size and statistical significance, across 515 tumors and 44 normal tissues. Expression was log2(expression + 1)-transformed, standardized within each gene, and truncated to -2.5 to 2.5; red and blue indicate relatively high and low expression. (B) Volcano plot of 936 metabolism-related genes. Axes show log2 fold change and -log10 Benjamini-Hochberg false discovery rate (FDR). Red, blue, and grey indicate upregulated, downregulated, and nonsignificant genes. At |logFC| > 0.5 and FDR < 0.05, 485 genes were differentially expressed (291 upregulated and 194 downregulated).

**Figure 3:**
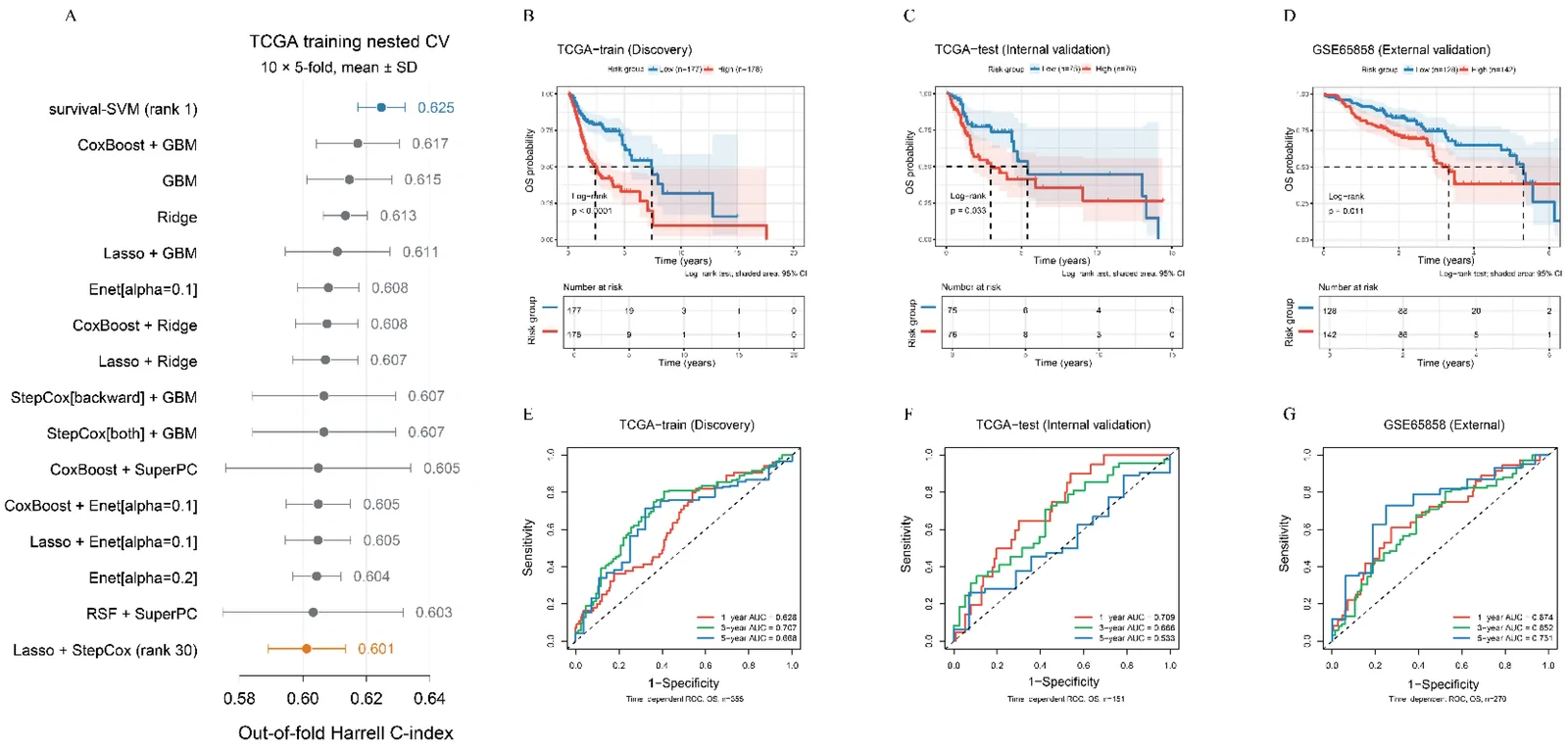
** Development and prognostic evaluation of the eight-gene risk score.** (A) Training-only repeated nested cross-validation in the TCGA training set (n = 355; 132 events), using 10 repeats of stratified five-fold cross-validation. Feature selection and hyperparameter tuning were performed within each outer-training fold. Points and error bars indicate the mean ± standard deviation of Harrell C-indices calculated from pooled out-of-fold predictions. The 15 highest-ranked configurations and LASSO + StepCox (rank 30) are shown. Held-out cohorts were excluded from ranking. (B-D) Kaplan-Meier overall survival curves for the TCGA training set (n = 355), internal test set (n = 151), and GSE65858 (n = 270). The training-set median risk score (-0.03298) was used as the fixed cutoff in all cohorts. Shading indicates 95% confidence intervals; P values were calculated using two-sided log-rank tests. (E-G) Time-dependent ROC curves for 1-, 3-, and 5-year overall survival in the same cohorts.

**Figure 4:**
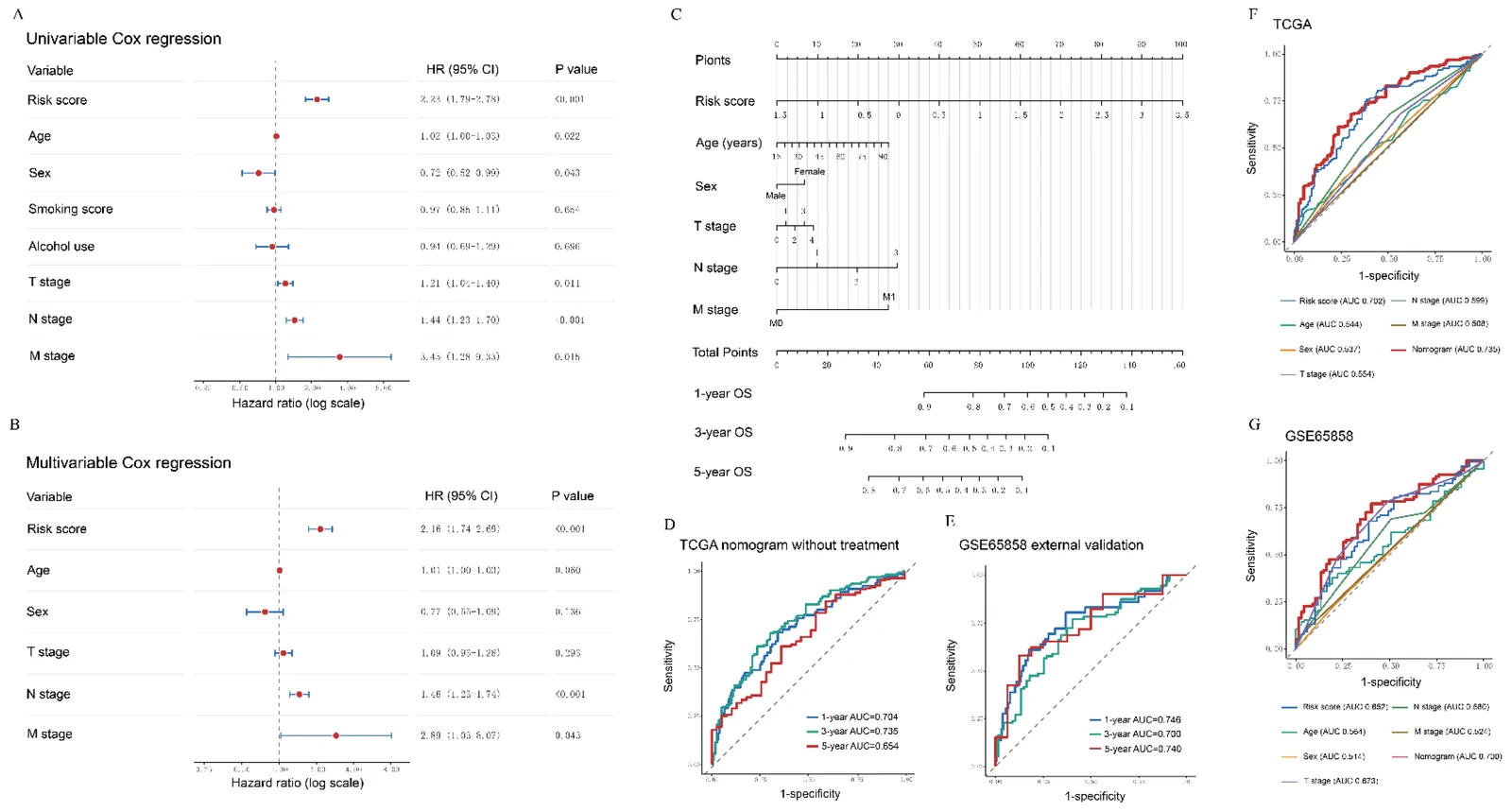
** Clinical prognostic model without treatment information.** (A, B) Univariable and multivariable Cox analyses in the complete-case TCGA cohort (n = 473, including 332 training and 141 internal test patients, with 174 deaths). Candidate variables included the risk score, age, sex, smoking score, alcohol use, and T, N, and M categories. Variables with an overall likelihood ratio test *p* < 0.05 in the univariable analysis entered the multivariable model. Points and horizontal lines indicate hazard ratios and 95% confidence intervals. Coefficient-level* p* values were calculated using two-sided Wald tests. Hazard ratios for age, T category, and N category were estimated per one-unit increase. Sex compares male with female, and M category compares M1 with M0. (C) Nomogram using the selected variables to predict 1-, 3-, and 5-year overall survival. Variables were not required to remain individually significant after multivariable adjustment. (D, E) Time-dependent ROC curves at 1-, 3-, and 5- years in TCGA and GSE65858. (F, G) 3-year ROC curves comparing the complete nomogram with individual predictors.

**Figure 5:**
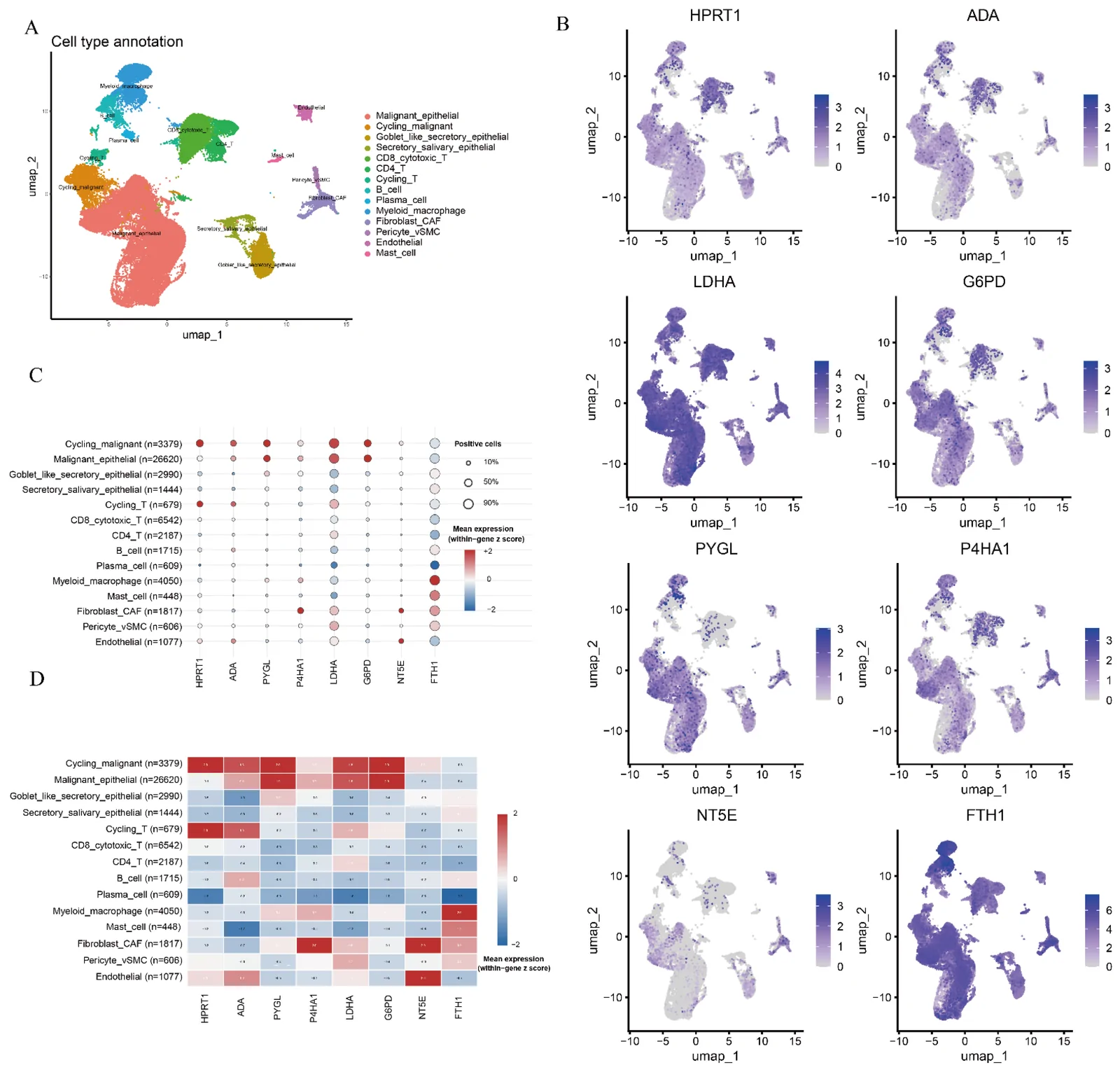
** Distribution of the eight model genes in the HNSCC single-cell microenvironment.** (A) Uniform manifold approximation and projection (UMAP) of 54,163 cells assigned to 14 cell populations. (B) Feature plots showing log-normalized RNA expression of HPRT1, ADA, PYGL, P4HA1, LDHA, G6PD, NT5E, and FTH1. A separate gene-specific color scale was used for each feature plot. Colors can therefore be compared spatially within a gene but not quantitatively between different genes. (C) Dot plot across cell populations. Dot size indicates the proportion of cells with expression > 0. Dot color represents mean expression standardized within each gene and truncated to -2 to 2. (D) Heatmap of cell-type mean expression using the same transformation and limits. Red and blue indicate relatively high and low expression within each gene.

**Figure 6:**
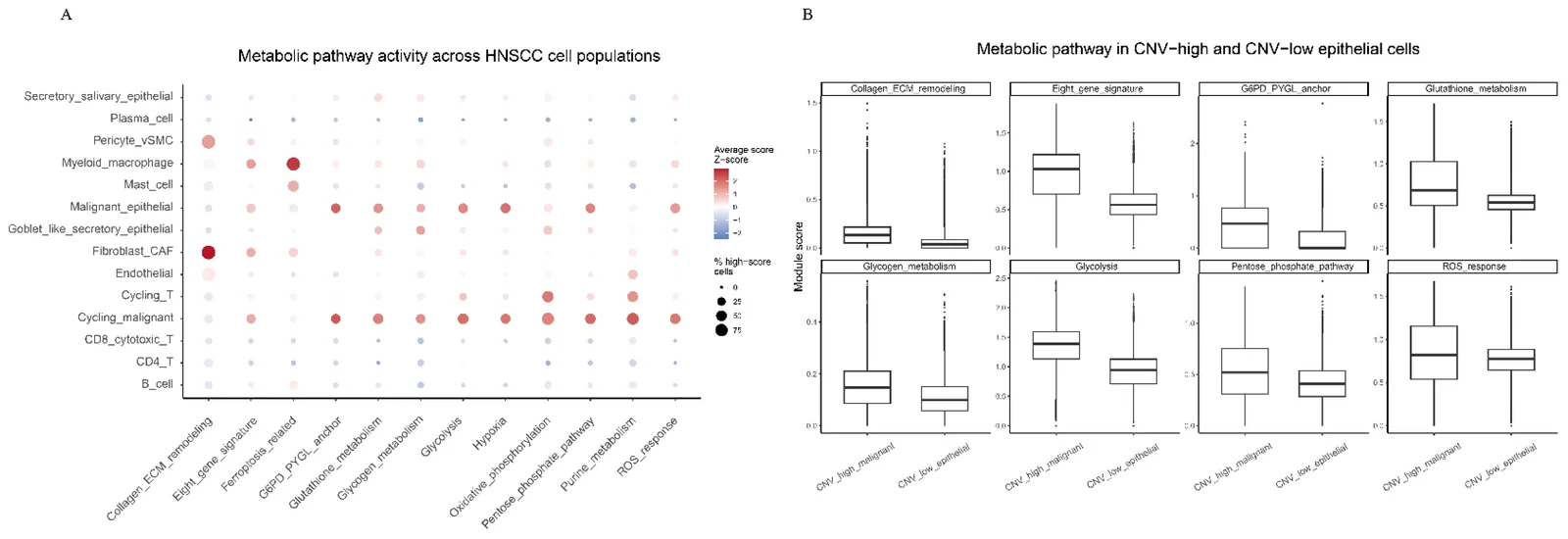
** Epithelial copy-number-variation states and prespecified metabolic modules.** (A) Dot plot of 12 prespecified modules: glycogen metabolism, the pentose phosphate pathway, glycolysis, oxidative phosphorylation, reactive oxygen species response, glutathione metabolism, ferroptosis-related genes, hypoxia, purine metabolism, collagen and extracellular matrix remodeling, the eight-gene signature, and the G6PD/PYGL anchor. Dot color represents the cell-type mean module score standardized across cell populations. Dot size represents the percentage of cells with a positive module score. (B) Cell-level distributions of eight selected module scores in CNV-high malignant epithelial cells (n = 16,240 cells) and CNV-low epithelial cells (n = 18,146 cells) from eight samples. Boxes show the median and interquartile range. Whiskers extend to 1.5 times the interquartile range, and points beyond the whiskers are shown as outliers. Comparisons were performed using two-sided Wilcoxon rank-sum tests at the cell level, followed by Benjamini-Hochberg correction. Individual cells were treated as the statistical units.

**Figure 7:**
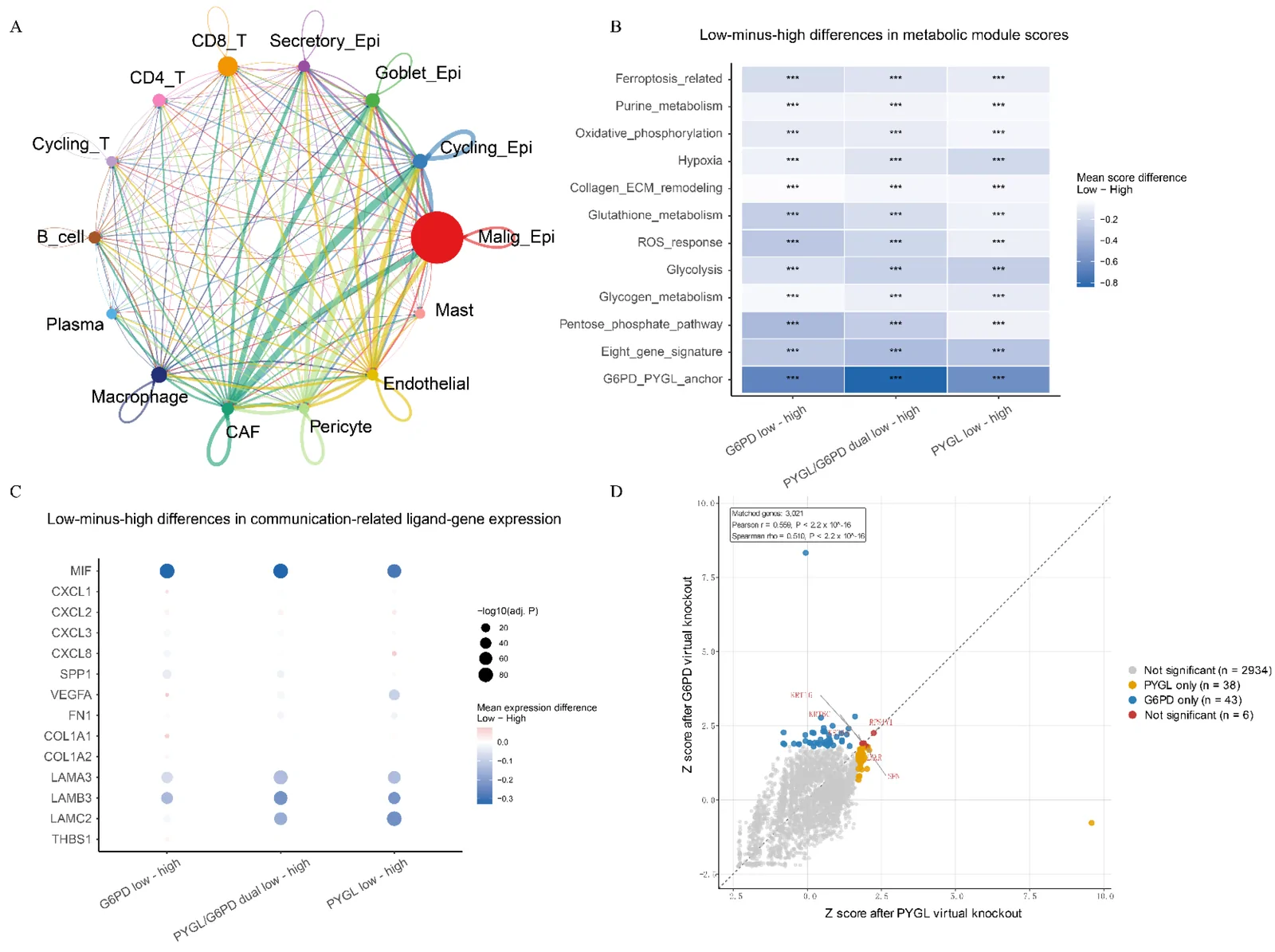
** Cell communication, expression-defined metabolic states, and computational perturbation analysis.** (A) CellChat network among 14 cell populations. Node size and color indicate cell number and population; directed edge width indicates communication strength, edge color identifies the sender, and self-loops indicate autocrine signaling. (B) Within-sample low-minus-high differences in 12 module scores for PYGL, G6PD, or combined PYGL/G6PD expression. Groups comprised 3,647 PYGL-high and 8,285 PYGL-low cells, 3,704 G6PD-high and 7,238 G6PD-low cells, and 4,063 dual-high and 5,734 dual-low cells. Negative values indicate lower scores in the low-expression group. Asterisks indicate Benjamini-Hochberg-adjusted *p* < 0.001 from cell-level Wilcoxon rank-sum tests. These observational contrasts do not represent gene knockout. (C) Low-minus-high differences in selected CellChat ligand-gene expression. Color shows the mean difference; size shows -log10(adjusted *p*). (D) Gene-level perturbation Z scores after scTenifoldKnk virtual knockout of PYGL and G6PD. The dashed line indicates equal scores. Grey points were nonsignificant; yellow, blue, and red indicate PYGL-specific, G6PD-specific, and shared significance at adjusted *p* < 0.05. Among 3,021 matched genes, 38 were PYGL-specific, 43 G6PD-specific, and 6 shared.

**Figure 8:**
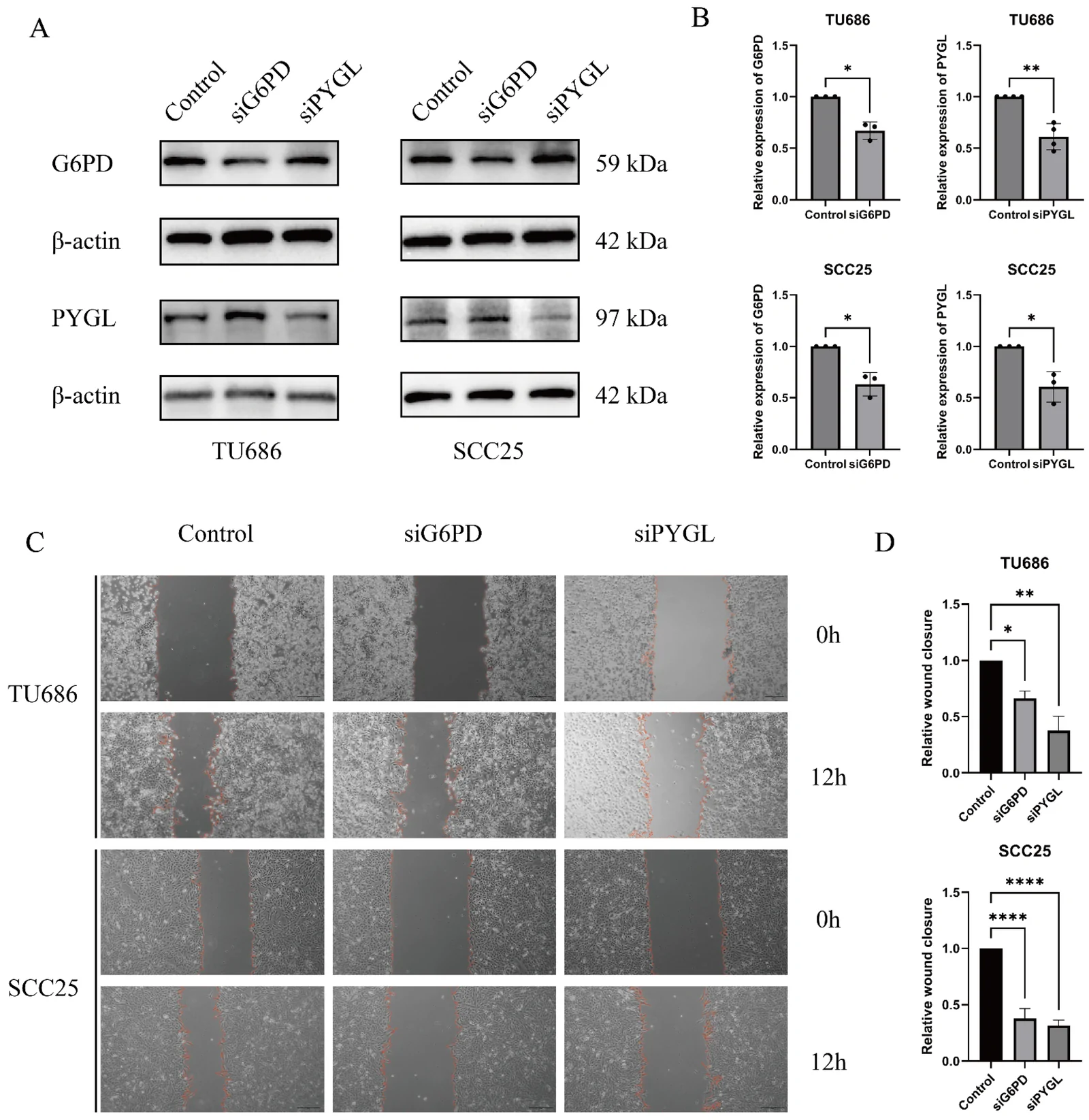
** Effects of PYGL or G6PD silencing on target protein expression and wound closure in HNSCC cells.** (A) Western blots of G6PD (59 kDa) and PYGL (97 kDa) in TU686 and SCC25 cells transfected with negative-control small interfering RNA (Control), siG6PD, or siPYGL. β-actin (42 kDa) was used as the loading control. (B) Target protein expression normalized to β-actin and Control. Data are mean ± standard deviation, and dots represent independent experiments. Two-sided paired t-test *p* values respectively were 0.0215, 0.0091, 0.0299, and 0.0437 for TU686 G6PD, TU686 PYGL, SCC25 G6PD, and SCC25 PYGL. (C) Wound-healing images obtained at 0 and 12 h. Red tracings indicate the wound boundaries. Scale bar, 500 μm. (D) Relative wound closure at 12 h (mean ± standard deviation). One-way analysis of variance with Dunnett correction compared each knockdown with Control. Adjusted *p* values were 0.0424 (siG6PD) and 0.0078 (siPYGL) in TU686 cells and < 0.0001 for both comparisons in SCC25 cells. * p < 0.05, ** p < 0.01, *** p < 0.001, **** p < 0.0001.

**Table 1:** Comparison of Clinicopathological Characteristics Between the TCGA Training and Internal Validation Cohorts.

	Patients, No. (%)			
**Characteristics**	**Total (N=473, 100%)**	**TCGA training (N=332, 70.19%)**	**Internal validation (N=141, 29.81%)**	***p***
Age (years), median (IQR)	61.0 (53.0-68.0)	61.0 (53.0-68.0)	61.0 (54.0-69.0)	0.775
Sex				0.952
Female	125 (26.4%)	88 (26.5%)	37 (26.2%)	
Male	348 (73.6%)	244 (73.5%)	104 (73.8%)	
Smoking status				0.809
No	104 (22.0%)	72 (21.7%)	32 (22.7%)	
Yes	369 (78.0%)	260 (78.3%)	109 (77.3%)	
Alcohol status				0.668
No	151 (31.9%)	104 (31.3%)	47 (33.3%)	
Yes	322 (68.1%)	228 (68.7%)	94 (66.7%)	
Overall stage				0.933
I-II	26 (6.2%)	18 (6.2%)	8 (6.0%)	
III-IV	396 (93.8%)	271 (93.8%)	125 (94.0%)	
T stage				0.277
T0	1 (0.2%)	0 (0.0%)	1 (0.7%)	
T1	52 (11.0%)	41 (12.3%)	11 (7.8%)	
T2	140 (29.6%)	97 (29.2%)	43 (30.5%)	
T3	102 (21.6%)	74 (22.3%)	28 (19.9%)	
T4	178 (37.6%)	120 (36.1%)	58 (41.1%)	
N stage				0.958
N0	199 (42.1%)	140 (42.2%)	59 (41.8%)	
N1	84 (17.8%)	59 (17.8%)	25 (17.7%)	
N2	180 (38.1%)	125 (37.7%)	55 (39.0%)	
N3	10 (2.1%)	8 (2.4%)	2 (1.4%)	
M stage				1.000
M0	468 (98.9%)	328 (98.8%)	140 (99.3%)	
M1	5 (1.1%)	4 (1.2%)	1 (0.7%)	
Pathological grade				0.095
G1	57 (12.6%)	43 (13.4%)	14 (10.5%)	
G2	277 (61.0%)	200 (62.3%)	77 (57.9%)	
G3	114 (25.1%)	72 (22.4%)	42 (31.6%)	
G4	6 (1.3%)	6 (1.9%)	0 (0.0%)	
Treatment modality				0.943
Surgery only	186 (39.3%)	132 (39.8%)	54 (38.3%)	
Surgery + radiotherapy	128 (27.1%)	89 (26.8%)	39 (27.7%)	
Surgery + chemotherapy	5 (1.1%)	3 (0.9%)	2 (1.4%)	
Surgery + chemoradiotherapy	153 (32.3%)	107 (32.2%)	46 (32.6%)	
Concurrent chemoradiotherapy	1 (0.2%)	1 (0.3%)	0 (0.0%)	
Disease type				0.084
Hypopharynx	7 (1.5%)	2 (0.6%)	5 (3.5%)	
Larynx	112 (23.7%)	79 (23.8%)	33 (23.4%)	
Nasal cavity	0 (0.0%)	0 (0.0%)	0 (0.0%)	
Oral cavity	289 (61.1%)	206 (62.0%)	83 (58.9%)	
Oropharynx	64 (13.5%)	45 (13.6%)	19 (13.5%)	
Other/unknown	1 (0.2%)	0 (0.0%)	1 (0.7%)	

Data are presented as median (IQR) or n (%). P values were calculated using the Wilcoxon rank-sum test for age and the Pearson chi-square test or Fisher exact test for categorical variables. Percentages are based on available cases; missing values were excluded from P-value calculations. TCGA smoking status was harmonized as never-smoker versus current/former smoker. Treatment modalities were retained from the original TCGA treatment detail field. Disease type was derived from the GDC TCGA-HNSC primary site field.

**Table 2:** Comparison of Clinicopathological Characteristics Between the Overall TCGA and GSE65858 Cohorts.

	Patients, No. (%)			
**Characteristics**	**Total(N=743, 100%)**	**TCGA overall(N=473, 63.66%)**	**GSE65858(N=270, 36.34%)**	***p***
Age (years), median (IQR)	60.0 (53.0-68.3)	61.0 (53.0-68.0)	58.6 (52.4-68.4)	0.192
Sex				0.005
Female	172 (23.1%)	125 (26.4%)	47 (17.4%)	
Male	571 (76.9%)	348 (73.6%)	223 (82.6%)	
Smoking status				0.171
No	152 (20.5%)	104 (22.0%)	48 (17.8%)	
Yes	591 (79.5%)	369 (78.0%)	222 (82.2%)	
Alcohol status				<0.001
No	182 (24.5%)	151 (31.9%)	31 (11.5%)	
Yes	561 (75.5%)	322 (68.1%)	239 (88.5%)	
Overall stage				<0.001
I-II	81 (11.7%)	26 (6.2%)	55 (20.4%)	
III-IV	611 (88.3%)	396 (93.8%)	215 (79.6%)	
T stage				0.918
T0	1 (0.1%)	1 (0.2%)	0 (0.0%)	
T1	87 (11.7%)	52 (11.0%)	35 (13.0%)	
T2	220 (29.6%)	140 (29.6%)	80 (29.6%)	
T3	160 (21.5%)	102 (21.6%)	58 (21.5%)	
T4	275 (37.0%)	178 (37.6%)	97 (35.9%)	
N stage				0.003
N0	293 (39.4%)	199 (42.1%)	94 (34.8%)	
N1	116 (15.6%)	84 (17.8%)	32 (11.9%)	
N2	312 (42.0%)	180 (38.1%)	132 (48.9%)	
N3	22 (3.0%)	10 (2.1%)	12 (4.4%)	
M stage				0.133
M0	731 (98.4%)	468 (98.9%)	263 (97.4%)	
M1	12 (1.6%)	5 (1.1%)	7 (2.6%)	
Treatment intensity				<0.001
Mono	264 (35.5%)	186 (39.3%)	78 (28.9%)	
Multi	476 (64.1%)	287 (60.7%)	189 (70.0%)	
Other	3 (0.4%)	0 (0.0%)	3 (1.1%)	
Disease type				<0.001
Hypopharynx	40 (5.4%)	7 (1.5%)	33 (12.2%)	
Larynx	160 (21.5%)	112 (23.7%)	48 (17.8%)	
Nasal cavity	0 (0.0%)	0 (0.0%)	0 (0.0%)	
Oral cavity	372 (50.1%)	289 (61.1%)	83 (30.7%)	
Oropharynx	166 (22.3%)	64 (13.5%)	102 (37.8%)	
Other/unknown	5 (0.7%)	1 (0.2%)	4 (1.5%)	

Data are presented as median (IQR) or n (%). Tests and missing-data handling are as described in Table 1. GSE65858 alcohol categories were harmonized as no reported consumption versus any reported consumption. Treatment codes mo0, multi, and palliative were mapped to Mono, Multi, and Other, respectively. Pathological grade was not compared because it was unavailable in GSE65858. Disease type was harmonized from GDC primary site for TCGA and the original subsite field for GSE65858.

**Table 3:** Comparison of Clinicopathological Characteristics Between the Overall TCGA and GSE117973 Cohorts.

	Patients, No. (%)			
**Characteristics**	**Total (N=550, 100%)**	**TCGA overall (N=473, 86.00%)**	**GSE117973 (N=77, 14.00%)**	***p***
Age (years), median (IQR)	61.0 (53.9-68.0)	61.0 (53.0-68.0)	60.9 (55.7-66.6)	0.998
Sex				0.419
Female	142 (25.8%)	125 (26.4%)	17 (22.1%)	
Male	408 (74.2%)	348 (73.6%)	60 (77.9%)	
Smoking status				0.077
No	128 (23.3%)	104 (22.0%)	24 (31.2%)	
Yes	422 (76.7%)	369 (78.0%)	53 (68.8%)	
Alcohol status				0.001
No	190 (34.5%)	151 (31.9%)	39 (50.6%)	
Yes	360 (65.5%)	322 (68.1%)	38 (49.4%)	
T stage				0.470
T0	1 (0.2%)	1 (0.2%)	0 (0.0%)	
T1	59 (10.7%)	52 (11.0%)	7 (9.1%)	
T2	170 (30.9%)	140 (29.6%)	30 (39.0%)	
T3	119 (21.6%)	102 (21.6%)	17 (22.1%)	
T4	201 (36.5%)	178 (37.6%)	23 (29.9%)	
N stage				0.380
N0	235 (42.7%)	199 (42.1%)	36 (46.8%)	
N1	101 (18.4%)	84 (17.8%)	17 (22.1%)	
N2	204 (37.1%)	180 (38.1%)	24 (31.2%)	
N3	10 (1.8%)	10 (2.1%)	0 (0.0%)	
Pathological grade				0.002
G1	58 (10.9%)	57 (12.6%)	1 (1.3%)	
G2	323 (60.8%)	277 (61.0%)	46 (59.7%)	
G3	144 (27.1%)	114 (25.1%)	30 (39.0%)	
G4	6 (1.1%)	6 (1.3%)	0 (0.0%)	
Treatment modality				0.561
Surgery only (S)	211 (38.4%)	186 (39.3%)	25 (32.5%)	
Surgery + radiotherapy (S+RT)	151 (27.5%)	128 (27.1%)	23 (29.9%)	
Surgery + radiochemotherapy (S+CRT)	182 (33.1%)	153 (32.3%)	29 (37.7%)	
Other/not classifiable	6 (1.1%)	6 (1.3%)	0 (0.0%)	
Disease type				<0.001
Hypopharynx	10 (1.8%)	7 (1.5%)	3 (3.9%)	
Larynx	125 (22.7%)	112 (23.7%)	13 (16.9%)	
Nasal cavity	11 (2.0%)	0 (0.0%)	11 (14.3%)	
Oral cavity	307 (55.8%)	289 (61.1%)	18 (23.4%)	
Oropharynx	96 (17.5%)	64 (13.5%)	32 (41.6%)	
Other/unknown	1 (0.2%)	1 (0.2%)	0 (0.0%)	

Data are presented as median (IQR) or n (%). Tests and missing-data handling are as described in Table 1. Treatment was classified according to the original GSE117973 categories: S, S+RT, and S+CRT. TCGA surgery plus chemotherapy and concurrent chemoradiotherapy, which cannot be mapped directly to these categories, were retained as Other/not classifiable. Overall stage and M stage were not compared because they were unavailable in GSE117973. Disease type was harmonized from GDC primary_site for TCGA and the original subsite field for GSE117973.

## Data Availability

The data that support the findings of this study are available in The Cancer Genome Atlas (TCGA) at portal.gdc.cancer.gov and the Gene Expression Omnibus (GEO) at https://www.ncbi.nlm.nih.gov/geo/. These data were derived from the following resources available in the public domain: TCGA-HNSC Cohort, GEO Dataset GSE65858: https://www.ncbi.nlm.nih.gov/geo/query/acc.cgi?acc=GSE65858 GEO Dataset GSE117973: https://www.ncbi.nlm.nih.gov/geo/query/acc.cgi?acc=GSE117973 GEO Dataset GSE172577: https://www.ncbi.nlm.nih.gov/geo/query/acc.cgi?acc=GSE172577 GEO Dataset GSE150321: https://www.ncbi.nlm.nih.gov/geo/query/acc.cgi?acc=GSE150321

## Abbreviations

ANOVA: Analysis of variance
AUC: Area under the receiver operating characteristic curve
BP: Biological process
CAF: Cancer-associated fibroblast
CC: Cellular component
C-index: Concordance index
CI: Confidence interval
CNV: Copy number variation
CoxBoost: Cox proportional hazards boosting
DNA: Deoxyribonucleic acid
DSS: Disease-specific survival
ECL: Enhanced chemiluminescence
ECM: Extracellular matrix
Enet: Elastic net
FBS: Fetal bovine serum
FDR: False discovery rate
GDC: Genomic Data Commons
GEO: Gene Expression Omnibus
GO: Gene Ontology
HMM: Hidden Markov model
HNSCC: Head and neck squamous cell carcinoma
HPV: Human papillomavirus
HR: Hazard ratio
IQR: Interquartile range
LASSO: Least absolute shrinkage and selection operator
logFC: Log2 fold change
MF: Molecular function
MRDEGs: Metabolism-related differentially expressed genes
MSigDB: Molecular Signatures Database
NADPH: Nicotinamide adenine dinucleotide phosphate
OS: Overall survival
PBS: Phosphate-buffered saline
PCA: Principal component analysis
plsRCox: Partial least squares Cox regression
PPP: Pentose phosphate pathway
PVDF: Polyvinylidene fluoride
RNA: Ribonucleic acid
RNA-seq: RNA sequencing
ROC: Receiver operating characteristic
ROS: Reactive oxygen species
RPMI-1640: Roswell Park Memorial Institute 1640 medium
RSF: Random survival forest
S: Surgery alone
S+CRT: Surgery plus chemoradiotherapy
S+RT: Surgery plus radiotherapy
scRNA-seq: Single-cell RNA sequencing
SDS-PAGE: Sodium dodecyl sulfate-polyacrylamide gel electrophoresis
siNC: Negative-control small interfering RNA
siRNA: Small interfering RNA
StepCox: Stepwise Cox regression
SuperPC: Supervised principal components
Survival-SVM: Support vector machine for survival analysis
TCGA: The Cancer Genome Atlas
TCGA-HNSC: TCGA Head and Neck Squamous Cell Carcinoma project
TNM: Tumor-node-metastasis
UMAP: Uniform manifold approximation and projection
vSMC: Vascular smooth muscle cell
