# Supplementary material for: An Eight-Gene Metabolic Prognostic Signature in Head and Neck Squamous Cell Carcinoma: Cross-Cohort Evaluation, Single-Cell Analysis, and Preliminary Functional Evaluation of PYGL and G6PD
Source: J Cancer. 2026 Oct 12;17(11):1895–914. doi: 10.7150/jca.134402 (PMC13686788; doi:10.7150/jca.134402)
Supplement: Supplementary file 1 [file jcav17p1895s1.pdf]

Supplementary Figures

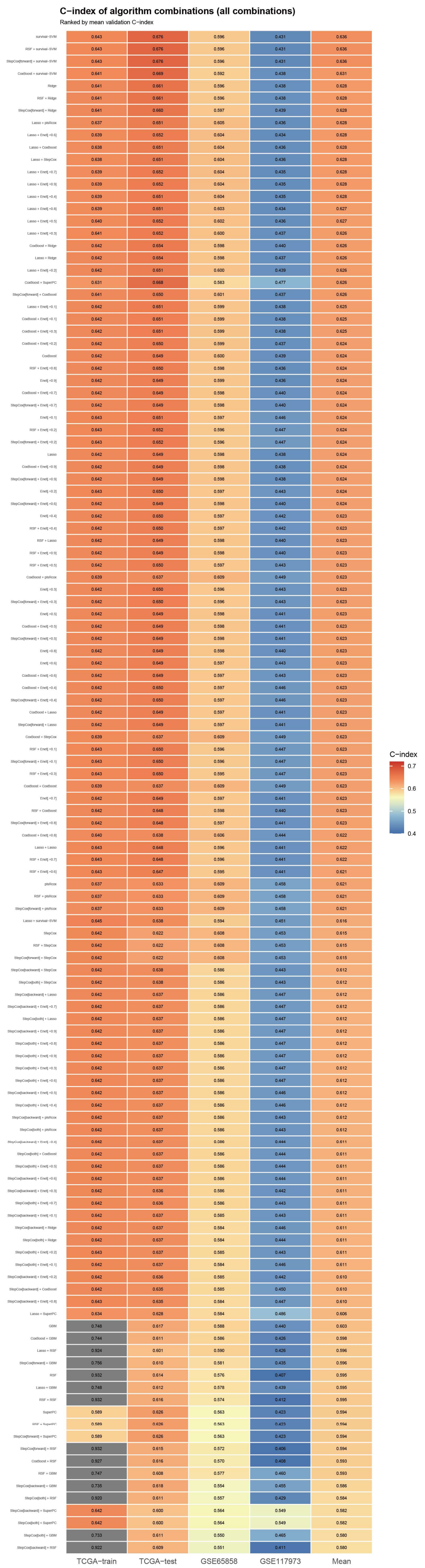
C−index of algorithm combinations (all combinations)
Ranked by mean validation C−index
TCGA−train
TCGA−test
GSE65858
GSE117973
Mean
C−index
0.7
0.6
0.5
0.4

Figure S1. Performance comparison of the machine-learning model combinations. The heatmap shows the concordance index (C-index) of the 124 algorithm combinations that were successfully fitted. Model performance was evaluated in the The Cancer Genome Atlas (TCGA) training cohort, TCGA internal test cohort, GSE65858 cohort, and GSE117973 cohort. The mean C-index across the evaluated cohorts is shown in the last column. Each cell contains the corresponding C-index, and the fill color indicates its magnitude. Model combinations are ordered according to their mean validation C-index.

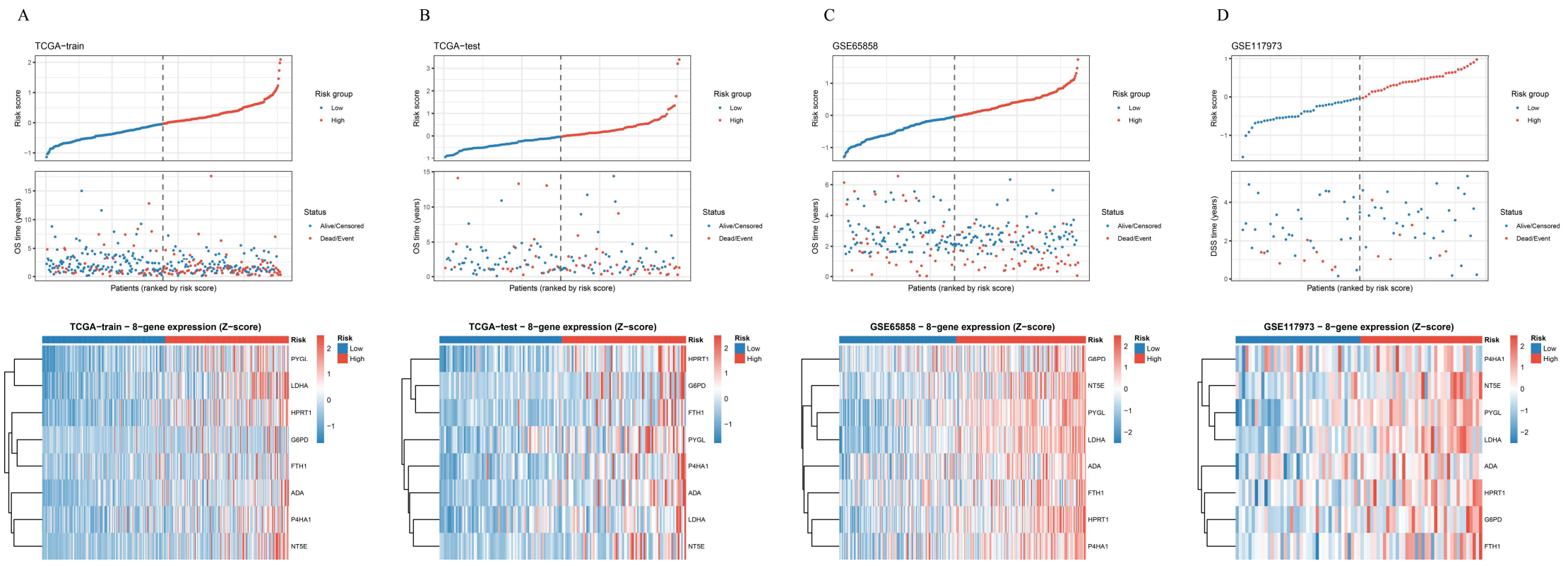

Figure S2. Distribution of risk scores, survival outcomes, and eight-gene expression profiles across the study cohorts. (A) TCGA training cohort. (B) TCGA internal test cohort. (C) GSE65858 cohort. (D) GSE117973 cohort. In each panel, the upper plot shows the distribution of the eight-gene risk score, the middle plot shows survival time and survival status, and the lower heatmap shows the expression profiles of HPRT1, ADA, PYGL, P4HA1, LDHA, G6PD, NT5E, and FTH1. Gene expression was standardized within each cohort using gene-wise Z scores. Patients were ordered by increasing risk score. The median risk score derived from the TCGA training cohort, −0.03298, was prespecified as the cutoff and applied unchanged to all other cohorts. Blue and red indicate the low-risk and high-risk groups, respectively. Overall survival was used as the endpoint in the TCGA and GSE65858 cohorts, whereas disease-specific survival was used in GSE117973.

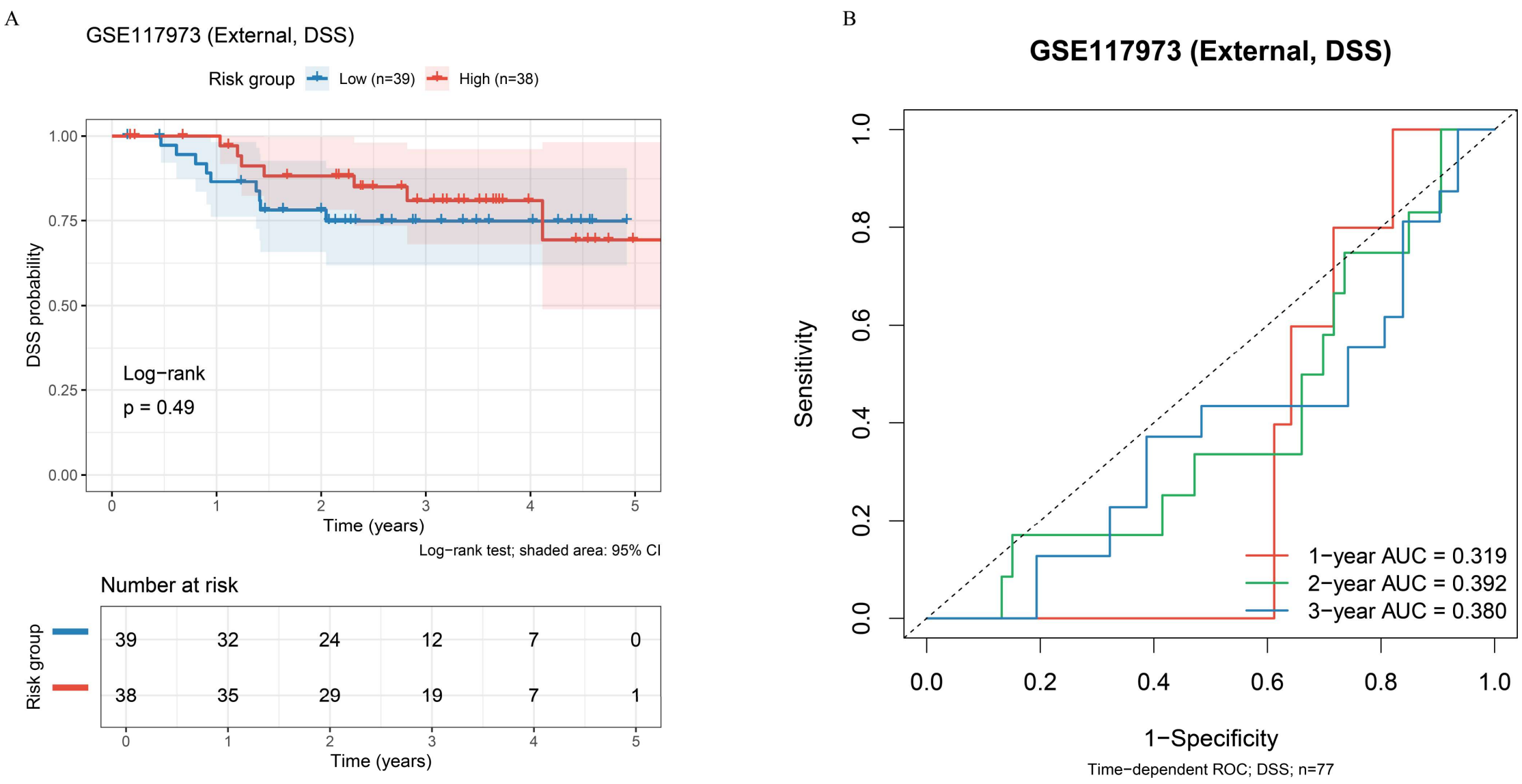

Figure S3. Exploratory evaluation of the eight-gene risk score in GSE117973. (A) Kaplan–Meier curves for disease-specific survival according to the risk groups defined using the prespecified TCGA training-cohort cutoff. The low-risk and high-risk groups contained 39 and 38 patients, respectively. Shaded areas indicate 95% confidence intervals. The difference between the groups was assessed using the log-rank test (p = 0.49). (B) Time-dependent receiver operating characteristic curves for predicting 1-, 2-, and 3-year disease-specific survival in 77 patients. The corresponding areas under the curve were 0.319, 0.392, and 0.380, respectively. The risk score had a C-index of 0.436 and was not significantly associated with disease-specific survival (hazard ratio = 0.807, 95% confidence interval: 0.323–2.013; p = 0.645).

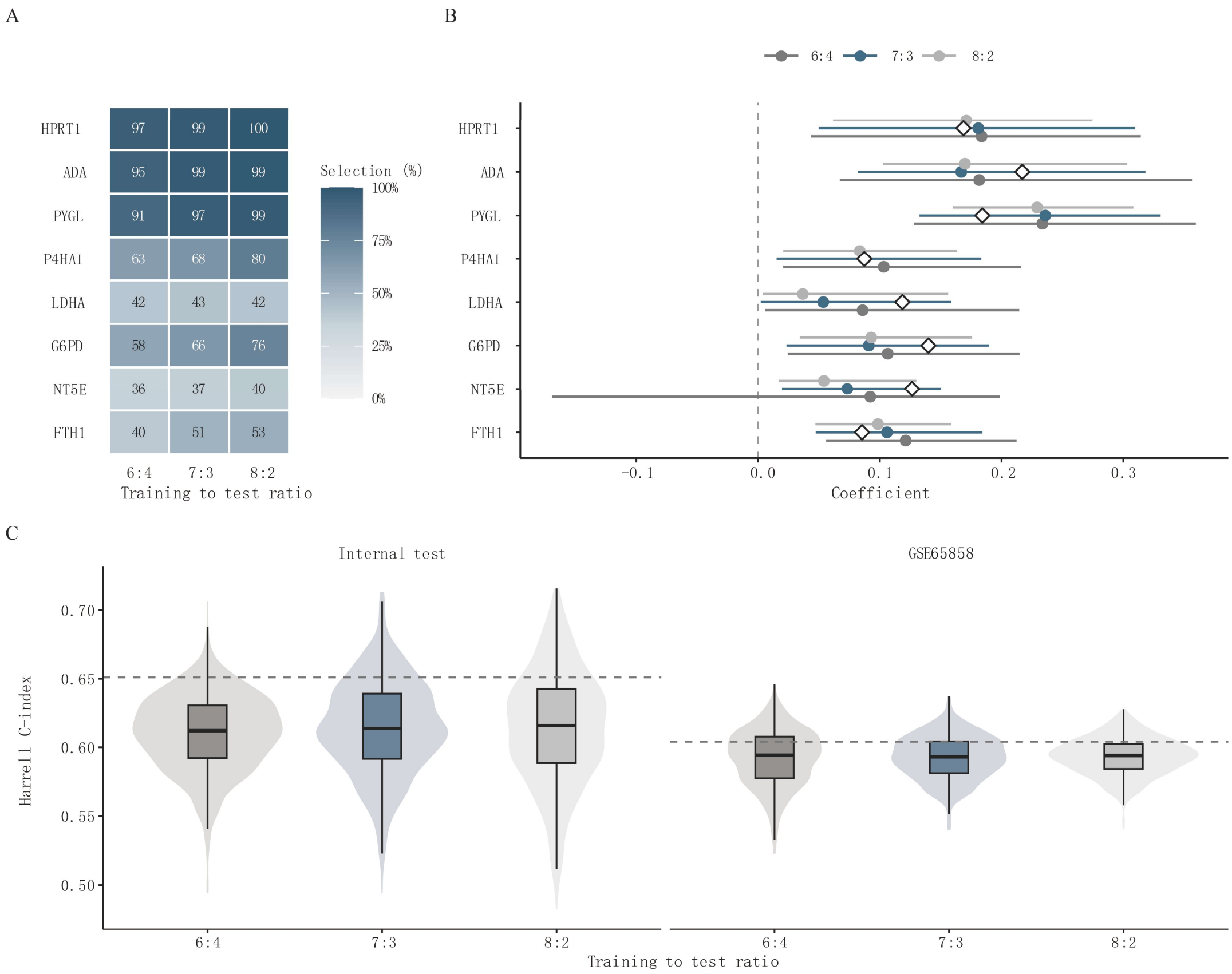

Figure S4. Sensitivity of the LASSO + StepCox model to training-to-test allocation. (A) Selection frequencies of the eight signature genes across repeated 6:4, 7:3, and 8:2 splits. (B) Coefficient distributions among successful repetitions; points and lines indicate medians and 95% intervals, and open diamonds indicate the coefficients of the original model. (C) Harrell C-index distributions in the internal test set and GSE65858; dashed lines indicate the performance of the original model. Each split ratio was evaluated in 500 outcome-stratified repetitions.

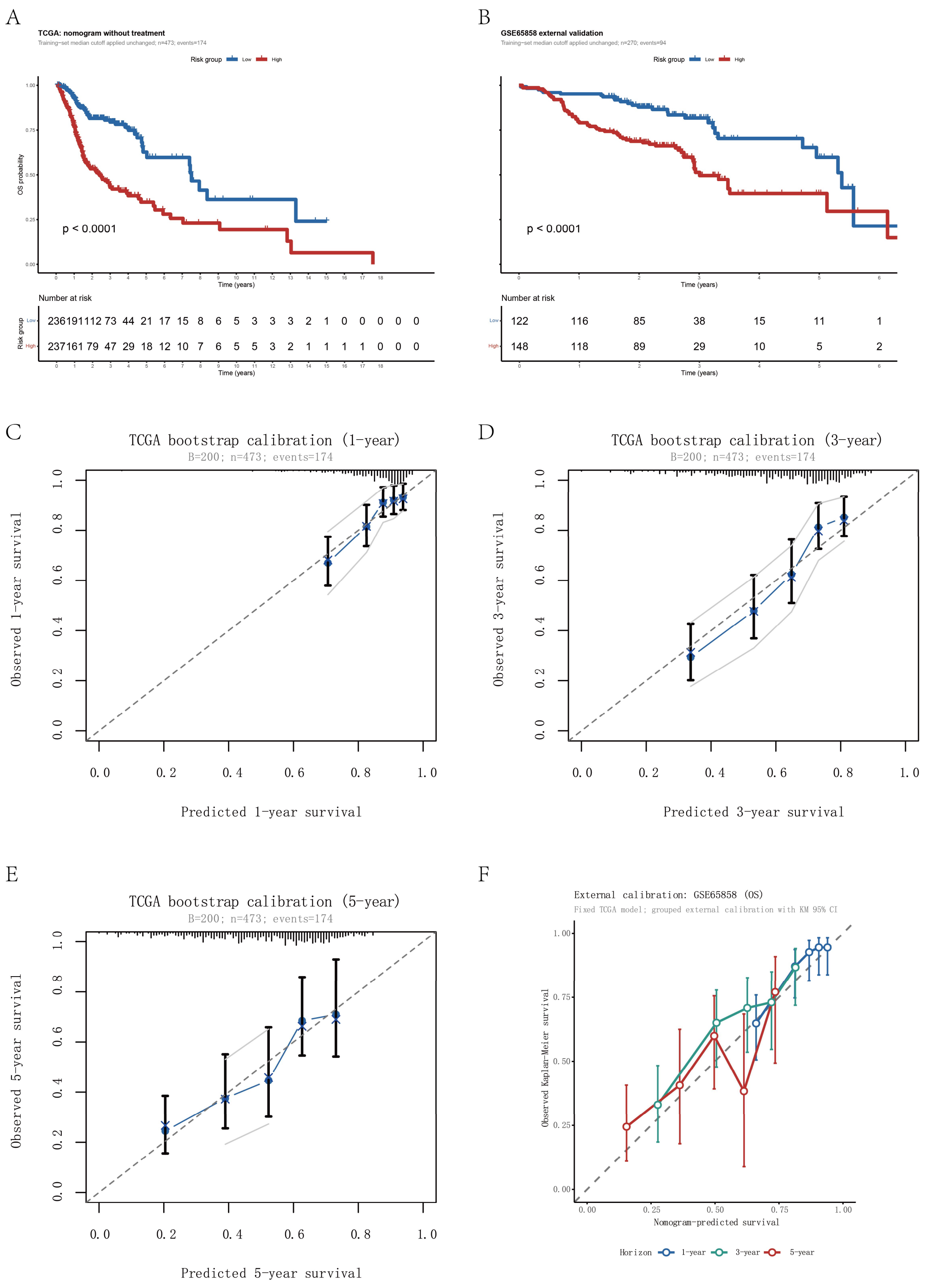

Figure S5. Survival stratification and calibration of the clinical prognostic model without treatment information. (A) Kaplan–Meier analysis of overall survival in the TCGA cohort using the median linear predictor from the TCGA model as the cutoff. The analysis included 473 patients and 174 deaths, with 236 patients in the low-risk group and 237 in the high-risk group. (B) External evaluation in GSE65858 using the same cutoff derived from TCGA without recalibration. The analysis included 270 patients and 94 deaths, with 122 patients in the low-

risk group and 148 in the high-risk group. Differences were evaluated using the log-rank test. (C–E) Bootstrap calibration curves for 1-, 3-, and 5-year overall survival in TCGA. Internal calibration was performed using 200 bootstrap resamples. (F) External calibration of the fixed TCGA model for 1-, 3-, and 5-year overall survival in GSE65858. Observed survival probabilities were estimated using the Kaplan–Meier method. Error bars represent Kaplan–Meier 95% confidence intervals, and the diagonal dashed line represents perfect agreement between predicted and observed survival.

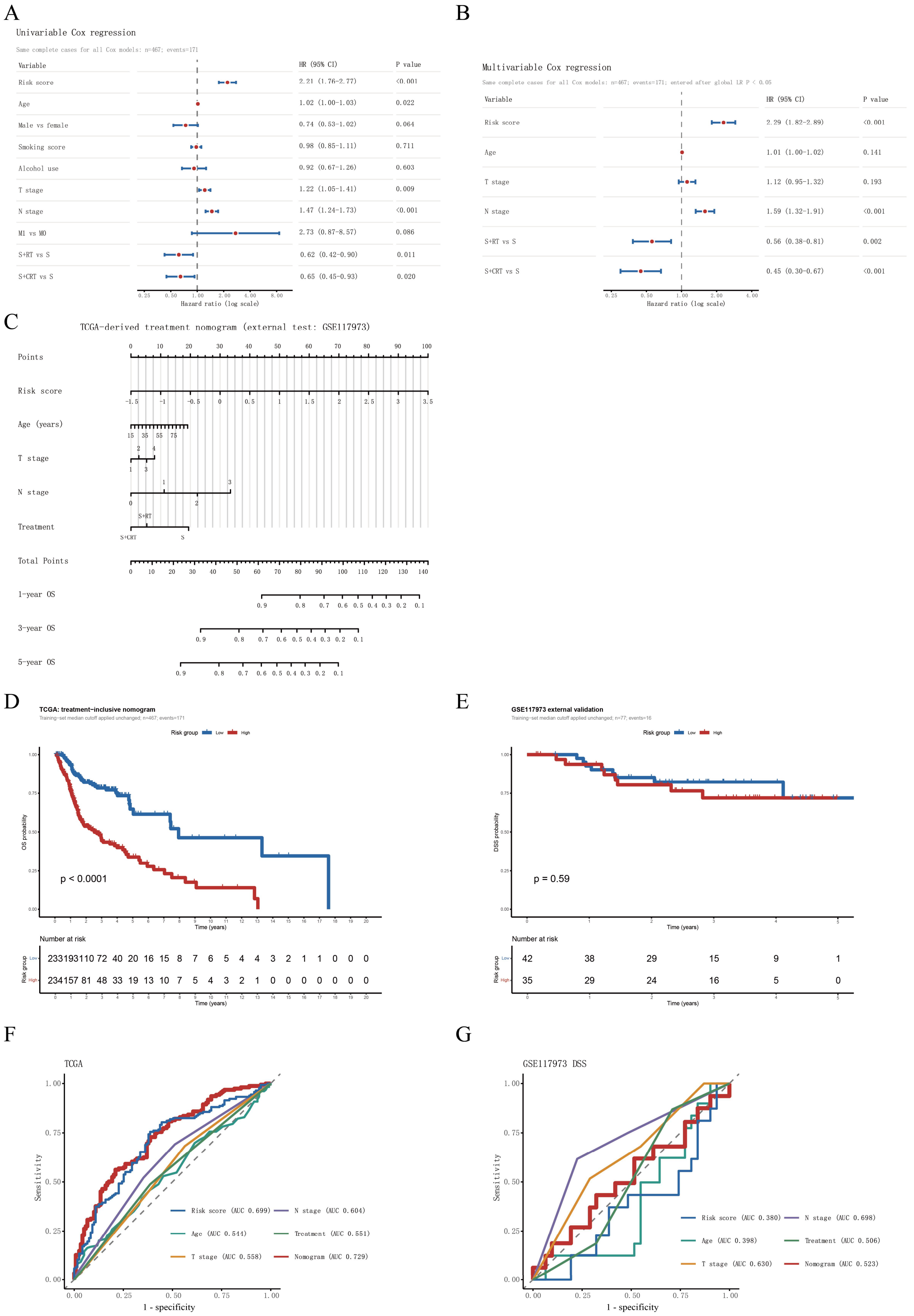

Figure S6. Development and exploratory evaluation of the treatment-extended clinical prognostic model. (A) Univariable Cox proportional hazards regression analysis in the TCGA complete-case population. The evaluated variables included the eight-gene risk score, age, sex, smoking history, alcohol consumption, T category, N category, M category, and treatment. Treatment was classified as surgery alone (S), surgery plus radiotherapy (S+RT), or

surgery plus chemoradiotherapy (S+CRT), with S as the reference category. (B) Multivariable Cox analysis performed in the same complete-case population. Variables with a global likelihood-ratio test P value below 0.05 in the univariable analysis were entered into the multivariable model. Both analyses included 467 patients and 171 deaths. Squares indicate hazard-ratio estimates, horizontal lines indicate 95% confidence intervals, and the vertical reference line indicates a hazard ratio of 1. (C) TCGA-derived nomogram incorporating the risk score, age, T category, N category, and treatment to estimate 1-, 3-, and 5-year overall survival probabilities. (D) Kaplan–Meier curves for the TCGA cohort stratified by the treatment-extended model. The low-risk and high-risk groups included 233 and 234 patients, respectively. (E) Exploratory Kaplan–Meier evaluation in GSE117973, including 77 patients and 16 disease-specific deaths. The low-risk and high-risk groups included 42 and 35 patients, respectively. (F) Comparison of 3-year time-dependent receiver operating characteristic curves in TCGA. The areas under the curve were 0.699 for the risk score, 0.544 for age, 0.558 for T category, 0.604 for N category, 0.551 for treatment, and 0.729 for the complete nomogram. (G) Exploratory comparison of 3-year disease-specific survival receiver operating characteristic curves in GSE117973. The areas under the curve were 0.380 for the risk score, 0.398 for age, 0.630 for T category, 0.698 for N category, 0.506 for treatment, and 0.523 for the complete nomogram.

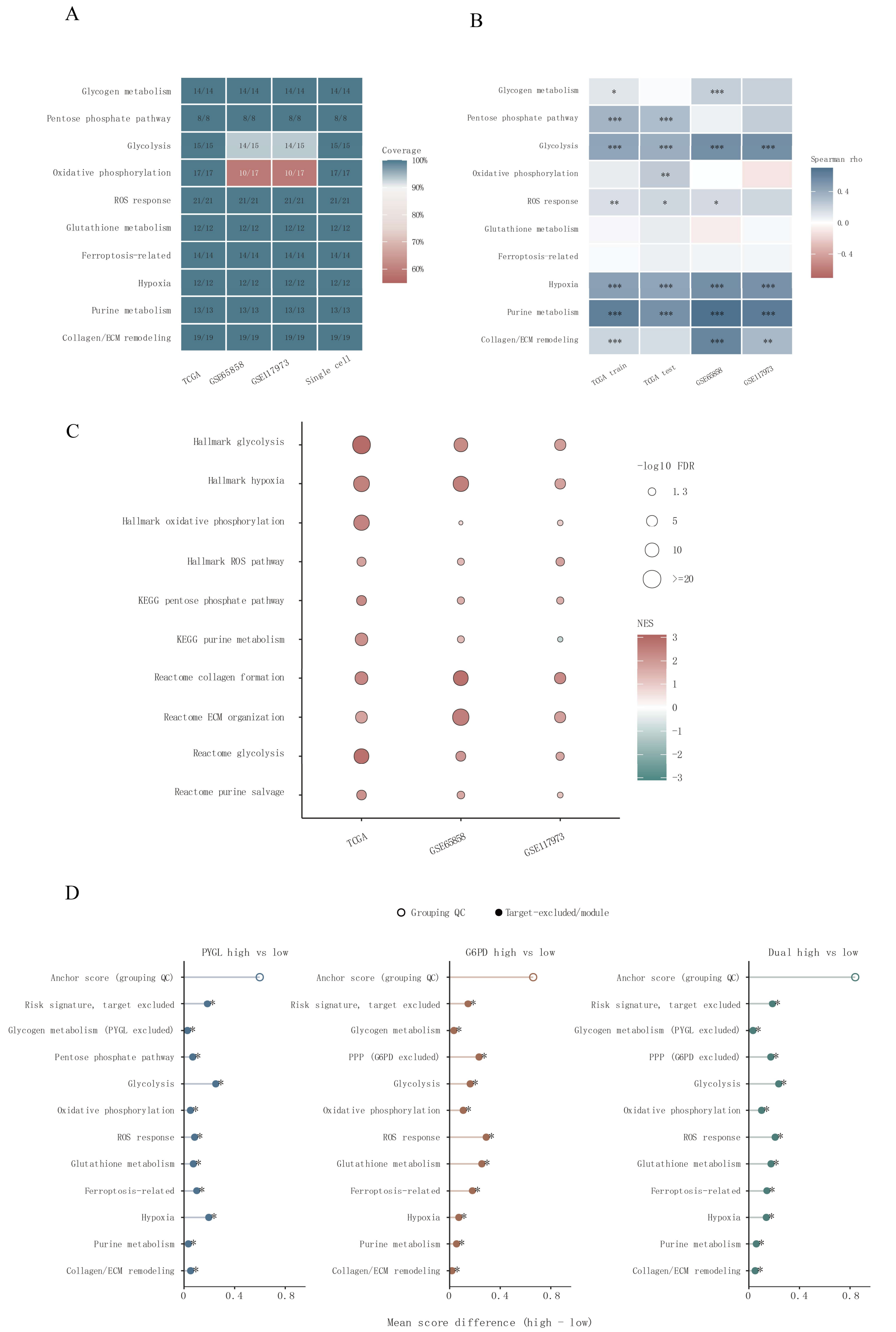

Figure S7. Pathway-resolved metabolic analysis and target-excluded sensitivity analysis. (A) Gene coverage of the 10 prespecified pathways across the bulk and single-cell datasets. (B) Spearman correlations between standardized ssGSEA pathway scores and the fixed eight-gene risk score. (C) Gene set enrichment analysis

comparing the fixed high- and low-risk groups; color indicates the normalized enrichment score and dot size indicates −log10(FDR). (D) Target-excluded comparisons of module scores between PYGL-, G6PD-, or dual-high and corresponding low-expression cells. Open points show the anchor score as grouping quality control. Asterisks indicate Benjamini–Hochberg FDR < 0.05.

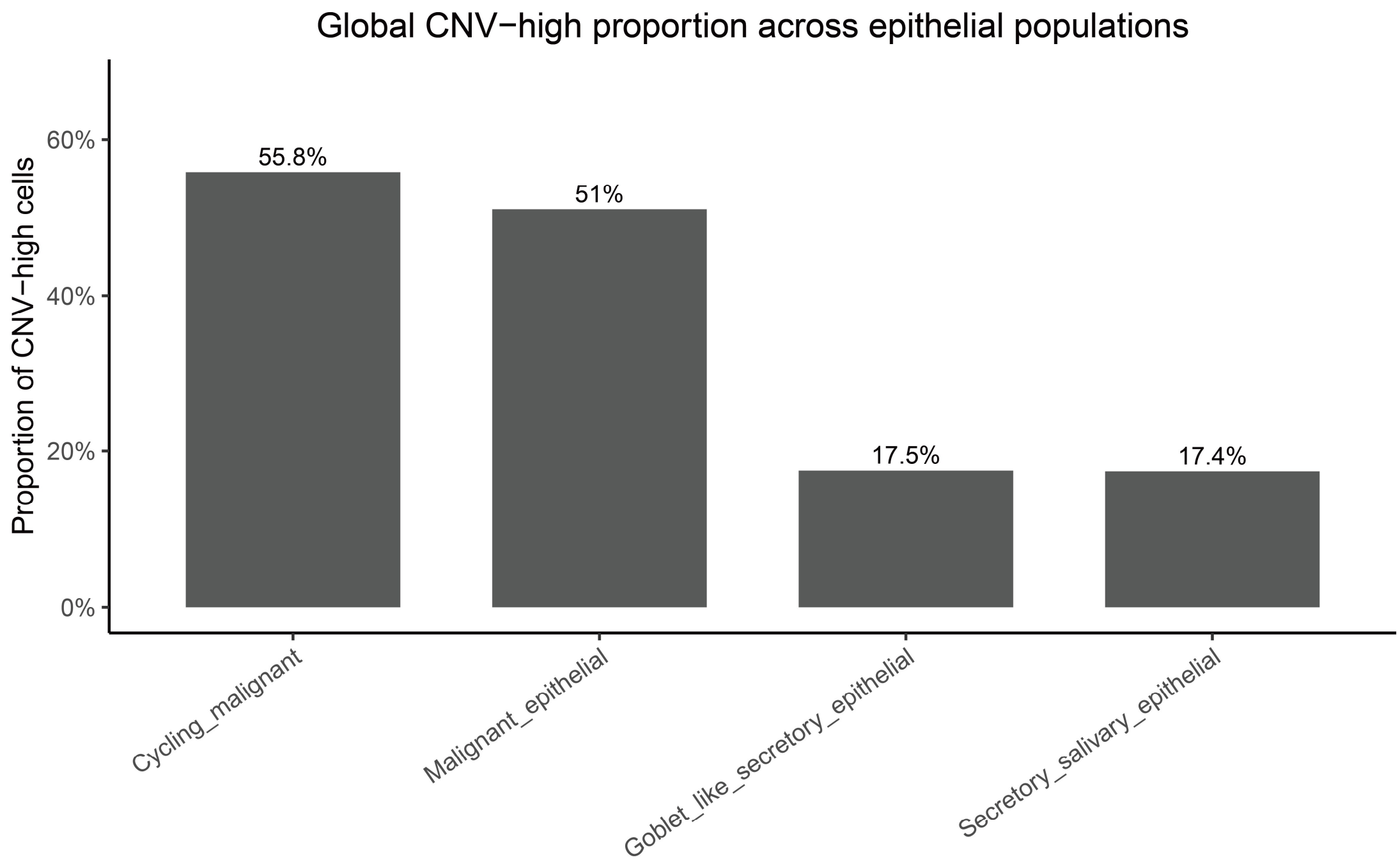

Figure S8. Global proportions of cells with high inferred copy-number variation across epithelial populations. The bar chart shows the proportions of cells classified as having high inferred copy-number variation (CNV) in four epithelial populations. The proportions were 55.8% in Cycling_malignant cells, 51.0% in Malignant_epithelial cells, 17.5% in Goblet_like_secretory_epithelial cells, and 17.4% in Secretory_salivary_epithelial cells.

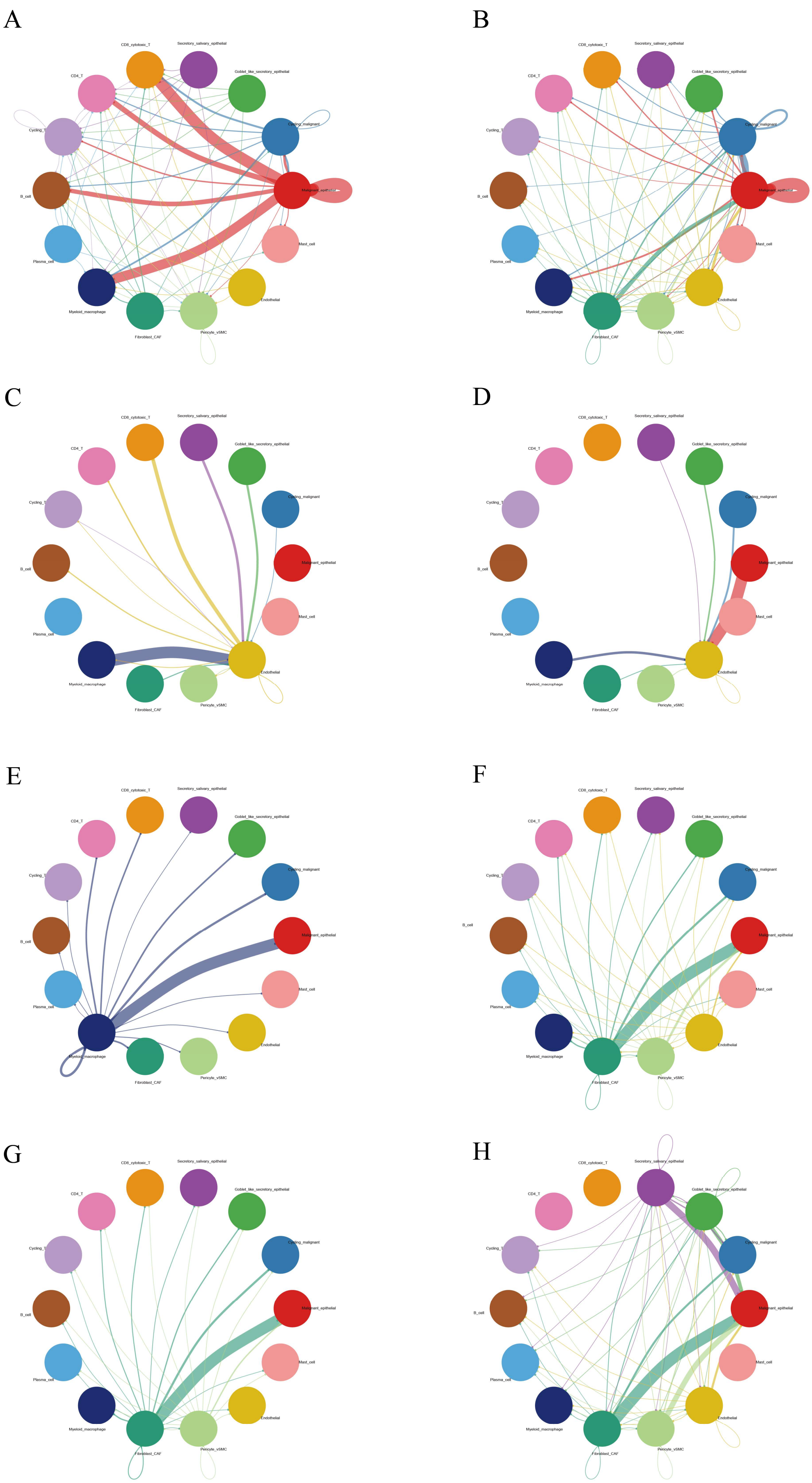
A
B
C
D
E
F
G
H
CD8_cytotoxic_T
Secretory_salivary_epithelial
Goblet_like_secretory_epithelial
CD4_T
Cycling_malignant
Malignant_epithelial
B_cell
Mast_cell
Plasma_cell
Endothelial
Myeloid_macrophage
Fibroblast_CAF
Pericyte_vSMC

Figure S9. CellChat-inferred intercellular communication networks in the HNSCC tumor microenvironment. Circular network plots show the inferred intercellular communication mediated by (A) MIF, (B) LAMININ, (C) CXCL, (D) VEGF, (E) SPP1, (F) COLLAGEN, (G) FN1, and (H) THBS signaling pathways across 14 annotated cell populations. Nodes represent cell populations, and node colors distinguish the annotated cell types. Directed edges connect signaling-source populations with predicted recipient populations. Edge width indicates the inferred communication probability or interaction strength. The MIF network included interactions involving MIF–CD74/CD44, MIF–CD74/CXCR4, and MIF–ACKR3. CXCL signaling included interactions involving CXCL1, CXCL2, CXCL3, and CXCL8, whereas VEGF signaling was primarily directed toward endothelial cells. SPP1 signaling was predominantly associated with myeloid macrophages. COLLAGEN, FN1, LAMININ, and THBS networks mainly reflected stromal and cancer-associated fibroblast signaling.

Supplementary Tables

TableS1. Metabolism-related differentially expressed genes between TCGA-HNSC tumor and normal tissues

Differential expression was analyzed using limma in 515 tumor and 44 normal tissue samples. Genes with |logFC| > 0.5 and a Benjamini-Hochberg FDR < 0.05 are listed. Positive and negative logFC values indicate upregulation and downregulation in tumors, respectively. P.Value and adj.P.Val denote the raw and adjusted *p* values.

Table S2. Coefficients and Cox regression results for the eight-gene risk score

The model was fitted in the TCGA training set. Gene expression was Z-standardized within each cohort. The risk score was calculated as the sum of each standardized expression value multiplied by its coefficient. HR denotes hazard ratio, and CI denotes confidence interval.

Table S3. Proportional hazards tests for the clinical model without treatment information

The proportional hazards assumption was assessed using Schoenfeld residuals in the complete-case TCGA cohort. GLOBAL represents the overall test for the model.

Table S4. Performance of the clinical prognostic models across cohorts

The model without treatment information was evaluated for overall survival in TCGA and GSE65858. The treatment-inclusive model was evaluated for overall survival in TCGA and explored for disease-specific survival in GSE117973. C-index denotes Harrell's concordance index. HRs are reported per one-unit increase in the model linear predictor.

Table S5. Kaplan-Meier analyses using fixed TCGA nomogram cutoffs

For the model without treatment information, the TCGA cutoff of 1.2496 was applied to TCGA and GSE65858. For the treatment-inclusive model, the TCGA cutoff of 0.9644 was applied to TCGA and GSE117973. *P* values were calculated using the log-rank test.

Table S6. Metabolic functions of the eight genes included in the risk score

The coefficients are those used in the final risk-score formula. The functional categories summarize the main metabolic or microenvironmental processes associated with each gene. PPP denotes pentose phosphate pathway, and ECM denotes extracellular matrix.

Table S7. Metabolic module scores in CNV-high and CNV-low epithelial cells

Module scores were compared between 16,240 CNV-high and 18,146 CNV-low epithelial cells using two-sided Wilcoxon rank-sum tests. *P* values were adjusted using the Benjamini-Hochberg method. Values shown as zero are below numerical precision.

Table S8. CellChat interaction-count matrix across 14 cell populations

Rows indicate sender populations, and columns indicate receiver populations. Each value represents the number of significant ligand-receptor interactions inferred by CellChat for the corresponding cell-population pair.

Table S9. CellChat ligand-receptor interactions across 14 cell populations

The table lists the sender and receiver populations, ligand-receptor pairs, communication probabilities, *p* values, signaling pathways, interaction categories, and supporting evidence. All listed interactions had $p < 0.05$.

Table S10. Cell counts in PYGL-high, PYGL-low, G6PD-high, G6PD-low, dual-high, and dual-low groups

PYGL and G6PD expression was standardized within each sample. The highest and lowest quartiles defined the high- and low-expression groups. The dual score was calculated as the mean of the PYGL and G6PD Z scores. NA indicates cells not assigned to either group. These groups represent naturally occurring expression states rather than gene knockdown.

Table S11. Overlap of significant genes predicted after PYGL and G6PD virtual knockout

Genes with an adjusted p value < 0.05 were considered significant. Shared denotes genes identified after both virtual knockouts. PYGL_only and G6PD_only denote genes identified in only one analysis.

Table S12. Gene Ontology enrichment of genes predicted after PYGL and G6PD virtual knockout

Enrichment analysis included biological process, cellular component, and molecular function terms. The *p*-value and q-value cutoffs were 0.05 and 0.25, respectively. Shared_KO denotes enrichment based on the six genes shared by the PYGL and G6PD analyses.

The raw image. pdf displays the original image of the Western Blot in the article.

The Wound-healing assay.zip displays the original images of cell scratches
